# Guide for Optimization of Olive Leaf Extraction and Silver Nanoparticles Biosynthesis as an Initial Step for Pilot Plant Design

**DOI:** 10.1021/acsomega.4c04483

**Published:** 2024-06-17

**Authors:** Anna Wirwis, Zygmunt Sadowski

**Affiliations:** Department of Process Engineering and Technology of Polymer and Carbon Materials, Wroclaw University of Science and Technology, Wybrzeze Wyspianskiego 27, 50-370 Wrocław, Poland

## Abstract

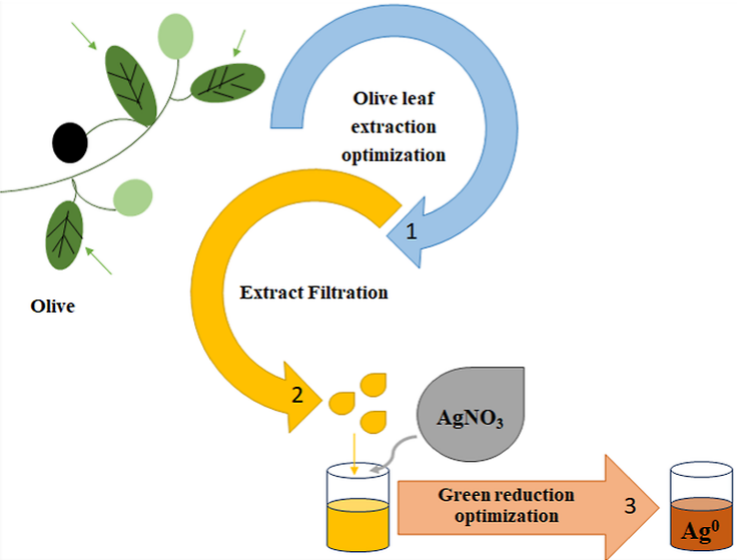

This account presents the results of two successful optimization processes. First, a polyphenol-rich aqueous olive extract was obtained and then silver nanoparticles (AgNPs) synthesized with high efficiency. Selected parameters for both processes were optimized based on the procedure of the Box–Behnken multifactorial design. The independent variables in the extraction process were the biomass/water ratio, temperature, and time. For AgNPs synthesis, the independent variables were the volume of olive extract, temperature, and process duration. The relationship between the process parameters was visualized graphically by using the response surface methodology. A high fit of the experimental data with the predicted models was shown. The regression coefficients were high, 0.9936 for extraction and 0.9757 for AgNPs biosynthesis. The extraction efficiency under its optimal conditions was as follows: biomass/solvent ratio 0.016, temperature 80 °C for 80 min, and yield 160.67 [μg GAE (gallic acid equivalent)/mL]. The highest yield of AgNPs synthesis, equal to 1.955, was obtained when it was carried out for 50 min at 75 °C with the application of 11 mL of extract. Studies on the AgNPs suspension’s stability depending on the extract amount were demonstrated. A physicochemical analysis using dynamic light scattering, transmission electron microscopy images, and Fourier transform infrared spectroscopy for AgNPs obtained under optimal conditions was shown. Finally, a pilot-scale biosynthesis of AgNPs was designed.

## 1 Introduction

In recent years, there has been growing interest in the biosynthesis of silver nanoparticles (AgNPs) using aqueous plant extracts.^1^ The advantages of this type of process lie in the low cost and easy access to renewable biomass, which is rich in many bioactive substances with reducing properties. Additionally, this process does not have a toxic effect on the environment. Several extracts prepared from various plant species have been traced and tested for the synthesis of AgNPs.^2,3^ According to Rodríguez Sousa and collaborators,^4^ olive trees are widely available in the Mediterranean region and occupy a total area of 6.8 million ha in Spain, Italy, Turkey, Portugal, and Greece. Therefore, using olive tree leaves as a cheap source of extraction raw material that contains a wealth of natural reducers for obtaining NPs is reasonable. Olive trees have many applications, such as the effective use of olive leaves in treating malaria symptoms or fighting fever.^5,6^ Extracts from the leaves of this plant have a diverse composition of polyphenols depending on the region in which they grow, with two main classes: secoiridoids and flavonoids.^7−10^ Additionally, they exhibit antioxidant,^11−13^ antimicrobial,^14−17^ and hepatoprotective properties.^18^ Conventional extraction methods for olive tree L. are good but are not beneficial when commercial applications are involved.^19^ Hence, using statistical modeling to reduce the costs and time of process optimization is essential.^12,20,21^ Alrugaibah and colleagues^21^ used a 75% aqueous ethanol solution to extract phenolic compounds from olive tree L. To optimize the extraction parameters, they applied an artificial neural network. Meanwhile, Oliveira et al. used the central composite design (CCD) to optimize olive leaf extraction.^12^ To improve the extraction effectiveness, plant leaves submerged in solvent are subjected to ultrasonic,^22−27^ microwave,^24,28−32^ or high-pressure exposure.^33^ The reducing properties of major polyphenols in the extract were used to synthesize NPs.^34,35^ The simplest method for synthesizing AgNPs involves directly combining a plant extract with a silver nitrate solution. The reducing substances contained in the extract will cause reduction of silver ions to metal NPs. In such a simple process, parameters such as the concentration of AgNO_3_ solution, the amount of extract used, pH, temperature, and reaction time play an important role.^36,37^ Optimization processes are becoming more popular in the biosynthesis of silver and other NPs, and the application of AgNPs depends on their specific size. Therefore, the optimization of NP formation focuses on obtaining the desired size.^38,39^ Process parameters were quantitatively evaluated via analysis of variance (ANOVA) for the synthesis of AgNPs using different plant extracts. For instance, eucalyptus extract^40^ was used to find optimal conditions for obtaining a minimal average size of AgNPs based on the statistical analysis of the interaction between silver nitrate concentration, time, plant extract concentration, and temperature of the synthesis. When using an extract from the leaves of a Mexican plant called purple heart, two types of responses—the smallest size of NPs and polydispersity index—were studied to optimize the biosynthesis of AgNPs according to the Box–Behnken design (BBD).^41^ In this case, the independent variables were AgNO_3_ concentration, temperature, and amount of plant extract. A similar procedure was adapted to optimize the parameters of the AgNP synthesis using *Boswellia sacra* leaf extract.^42^ The response of the measuring system was the absorbance value of 422 nm.

The main objective of the research presented here is to determine the optimal conditions for the effective extraction process of polyphenols from olive tree leaves (*Olea europaea* L.) and to optimize the synthesis of AgNPs using the resulting extract. The three-level BBD and response surface methodology (RSM) were used as tools for optimizing processes using biomass/solvent ratio, temperature, and time of extraction, as well as olive tree extract volume, temperature, and time of the synthesis as independent input variables for mathematical analyses, respectively. In both processes, a maximum surface response was sought.

As a final purpose, it was planned to design a pilot plant for biosynthesis of AgNPs according to optimized conditions for both steps. It was assumed that this would enable them to be used as the main building element of a specific nanomaterial with antibacterial properties, which is essential in the production of food storage packagings. This approach is a future alternative to harmful and environmentally polluting plastic packagings that are dangerous to the health of the population.

## 2 Materials and Methods

### 2.1 Materials

Olive tree leaves (*O. europaea* L.) are a popular commercially available plant material. Olive tree leaves were purchased in the form of cut and dried fragments, closed in a tight and opaque package. They were acquired in a store that is a distributor of various herbs and plants in Poland. Olive leaves were imported from the Mediterranean region. However, data related to the cultivation of the plant are not available. Quality tests for the selected plant material were not performed, while it was selected among all the plant leaves tested as one of the richest sources of polyphenols. Before the extraction process, the leaves were ground using a coffee grinder, to increase the efficiency of bioactive compound isolation. The grinding was performed for 1 min at the highest power setting on the grinder. The extraction solvent used was Milli-Q-ultrapure, double-distilled water with a high purity level. All other necessary reagents for the extraction process, standard curve preparation, and polyphenol content analysis were obtained from commercial sources. Folin and Ciocalteu’s phenol reagent and silver nitrate were purchased from Sigma-Aldrich, while acetic acid and sodium carbonate were obtained from POCH S.A. Gallic acid was received from Acros Organics.

### 2.2 Extraction of Polyphenols from Olive Tree Leaves’ Procedure

To extract the bioactive compounds, 100 mL of water was added to a selected sample of olive leaf biomass in a plastic vessel, which was then placed in a water bath heated to a specific temperature. After extraction, the biomass was separated from the extract by filtration using a 0.22 μm pore diameter Whatman filter (Sigma-Aldrich). To determine the efficiency of the extraction process, the Folin–Ciocalteau test was performed on each extracted sample according to the procedure known in the literature and used by us in previous studies.^43,44^ The absorbance values of each sample were measured at a wavelength of 750 nm using a UV–vis spectrophotometer and then converted to total polyphenol content (TPC) [μg GAE/mL] based on a calibration curve previously prepared using gallic acid as the standard substance. The regression coefficient of the standard curve with equation *y* = 0.003*x* – 0.0037 was 0.9979, and the equation for determining TPC is shown below (eq 1)
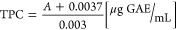
1where TPC [μg GAE/mL] represents the TPC, which is the response for the statistical optimization of olive leaf extraction, and *A* represents the spectrophotometrically measured absorbance value.

### 2.3 AgNPs Synthesis Procedure

The synthesis of NPs was generally carried out using the procedure from previous experiments.^44^ However, some modifications were related to the different synthesis conditions specified by the optimization process. Initially, a 10^–3^ M solution of silver nitrate in water was prepared and added in a volume of 50 mL to a flask containing a magnetic core, which was then protected from light with aluminum foil. Subsequently, a suitable volume of olive extract (obtained under optimal conditions using RSM) was placed in a dropping funnel also covered with foil. The extract was slowly added dropwise to the silver nitrate solution while constantly stirring. After the synthesis was completed, the mixture was heated at different temperatures for varying periods in a dark room to optimize the biosynthesis conditions of AgNPs. After the synthesis was completed, the mixture was cooled, and the entire solution was transferred to a dark glass bottle and kept at a low temperature of 4 °C in a fridge for further analysis.

It should be noted that silver compounds are highly sensitive to light. For this reason, in our case, the exposure of an aqueous solution of silver nitrate to light may lead to the reduction of Ag^+^ to a NP form (AgNPs). Because the aim of this work is to optimize the efficient biosynthesis of AgNPs using only natural reducers, polyphenols present in the water extract of olive leaves, additional reduction caused by light should be prevented by using aluminum foil, which covers each reaction vessel during this process. Additionally, the postreaction mixture is stored in a dark glass bottle. This treatment allows protection against light access because its presence during the designated storage period would lead to an additional reduction of unreacted Ag^+^ ions present in the solution or even accelerated destabilization of AgNPs as a result of their agglomeration and thus to inaccurate tests characterizing the physicochemical properties of AgNPs and their stability, especially in the perspective of long-term research.

### 2.4 Methods and Laboratory Equipment for AgNP Characterization

Several research techniques were employed to characterize the obtained AgNPs, including UV–vis spectrophotometry, dynamic light scattering (DLS) for NP analysis, the Fourier transform infrared (FTIR) spectroscopy method, and microscopic techniques. The usefulness and details of the equipment used for each method are described below.

#### 2.4.1 Ultraviolet–Visible Spectral Analysis

Ultraviolet–visible (UV–vis) spectral studies were conducted using a Shimadzu UV–vis 1900i spectrophotometer in a plastic cuvette with an optical path of 10 mm. The measurement was performed 1 day after synthesis in the 200–800 nm wavelength range, with simultaneous spectrum analysis using LabSolutions UV–vis software. This analytical method confirmed the formation of AgNPs, based on the appearance of a characteristic band in the spectrum in the wavelength range of 418–440 nm. Stability studies were conducted over time by using this method.

#### 2.4.2 DLS Analysis

The physicochemical characterization of the resulting AgNPs, including particle size distribution and hydrodynamic size value in a suspension, was analyzed using a diffraction light detector and Photocor Complex analyzer (Photocor). The measurement was performed the day after the NPs were synthesized at a temperature of 25 °C.

#### 2.4.3 Transmission Electron Microscopy Measurements

Transmission electron microscopy (TEM) images was obtained using the FEI Tecnai G2 20 X-TWIN high-resolution transmission electron microscope with the LaB6 cathode, FEI Eagle 2K CCD camera, EDS detector, and STEM detector. Before the measurement, the sample of AgNP suspension was applied to a copper measuring mesh covered with a carbon film and allowed to dry.

#### 2.4.4 FTIR Spectroscopy Analysis

FTIR analysis was performed for the aqueous olive leaf extract obtained under optimal conditions and for the sample formed with it after the reduction of Ag^+^ ions. Using this method, changes in the position of the bands corresponding to the functional groups of polyphenolic compounds were observed, which at the same time identified the appearance of interactions between them and the surface of the formed AgNPs. FTIR measurement was carried out using a Bruker Vertex 70 FTIR spectrometer, using a range of 4000–400 cm^–1^.

### 2.5 Box–Behnken Factorial Design

The optimization of two interrelated processes involved the extraction of polyphenols from olive leaves in an aqueous solvent and determination of optimal conditions for the efficient synthesis of AgNPs using olive extract. Both processes were optimized based on three selected parameters defined as independent variables. The Box–Behnken factorial design was used to assess the effect of each parameter on the maximum value of the responses. The design involved three levels (−1), (0), and (1), which were determined based on single-factor experiments in the first stage of the experimental plan. The factor-level values for the extraction process and for the synthesis of AgNPs are presented in Table 1.

**Table 1:** Three Levels of Optimization Parameters for the Extraction of Polyphenols from Olive Leaves and AgNP Biosynthesis Using a Box–Behnken Factorial Design

type of process	independent variable (uncoded units)	coded variable level			dependent variable (response)
		(−1)	(0)	(1)	
olive leaf extraction	biomass/solvent volume (*X*_1_)	0.008	0.012	0.016	TPC [μg GAE/mL] (TPC)
	temperature [°C] (*X*_2_)	50	70	90	
	time [min] (*X*_3_)	50	70	90	
AgNPs biosynthesis	extract volume [mL] (*X*_1_)	10	12.5	15	AgNPs yield (*A*)
	temperature [°C] (*X*_2_)	65	70	75	
	time [min] (*X*_3_)	40	45	50	

The actual values (uncoded) of the independent variables for both processes were converted to their coded equivalents, represented as (−1), (0), and (1) for each process parameter, using eq 2
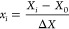
2where *x*_*i*_ represents the mean dimensionless values of the parameters for both processes, *X*_*i*_ represents the uncoded value of the three tested variables compatible with the process under consideration, *X*_0_ represents the uncoded value of each variable at the central point, and Δ*X* represents the mean step change of the value of the substantial variable (*X*_i_) corresponding to a variation of the coded value of process variables.

The Box–Behnken statistical model was used to optimize the extraction of bioactive polyphenolic compounds from olive leaves into the water. The model consisted of 17 combinations of input parameters, with the response represented by the TPC expressed in units of μg GAE/mL. Five experiments from the total were a series of extractions carried out under identical conditions, constituting the central points of the model, with a value at level 0 assumed for each parameter during the extraction. The ranges of levels for each independent variable and the values of the dependent variable (TPC) obtained as a result of the extraction of the plant material are presented in Table 2. Box–Behnken statistical design was employed to determine the most effective conditions for the biosynthesis of AgNPs using olive extract obtained under previously optimized conditions. The design matrix contained 14 variations between the synthesis conditions, including two repeated experiments, with conditions corresponding to the central point. Table 3 illustrates the values of uncoded and coded independent variables, including the extract volume, temperature, and time of synthesis of AgNPs, as well as the biosynthesis yields expressed as maximum absorbance values (*A*) from the UV–vis spectra, which are surface responses. The predicted response values were determined using a linear regression equation obtained as a result of statistical calculations, called the second-order polynomial and illustrated in the general form below (eq 3)
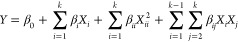
3where the individual components of the equation are as follows: *Y* represents the predicted response (TPC [μg GAE/mL] or yield of AgNP synthesis—*A* [absorbance value]), β_0_, β_*i*_, and β_*ii*_, β_*ij*_ are regression coefficients that correspond to variable correlations’ response, β_*i*_ represents the linear coefficients, β_*ii*_ represents the quadratic coefficients and β_*ij*_ represents the coefficients of cross-interactions between two independent input variables, *k* represents the number of all independent variables, and *X*_*i*_ and *X*_*j*_ represent the value of uncoded independent variables. A specialized program for statistical calculations, Statistica 13.3, was used to obtain a general regression equation, and then its final form was obtained by assessing the significance of the impact of individual process conditions and their correlation on the model and the response obtained using parameters such as *p*-value, *F*-value, lack of fit (LoF) value, and *R*^2^, *R*_adj_^2^, and *R*_pred_^2^, obtained by the ANOVA test.

**Table 2:** Box–Behnken Factorial Design with the Independent Variables and Experimental and Predicted TPC Values in Aqueous Olive Tree L. Extract^a^

run	coded variable			actual variable			TPC [μg GAE/mL]		
	*x*_1_ [g/mL]	*x*_2_ [°C]	*x*_3_ [min]	*X*_1_ [g/mL]	*X*_2_ [°C]	*X*_3_ [min]	actual	predicted	residual
1	–1	–1	0	0.008	50	70	97.13	94.96	2.17
2	1	–1	0	0.016	50	70	121.47	121.67	–0.20
3	–1	1	0	0.008	90	70	133.47	133.27	0.20
4	1	1	0	0.016	90	70	156.00	158.17	0.10
5	–1	0	–1	0.008	70	50	109.13	108.89	0.12
6	1	0	–1	0.016	70	50	139.80	137.20	–2,17
7	–1	0	1	0.008	70	90	126.23	128.84	–2.61
8	1	0	1	0.016	70	90	151.90	152.14	–0.24
9	0	–1	–1	0.012	50	50	93.23	95.64	–2,41
10	0	1	–1	0.012	90	50	134.23	134.66	–0.43
11	0	–1	1	0.012	50	90	115.13	114.70	0.43
12	0	1	1	0.012	90	90	152.90	150.49	2.41
13	0	0	0	0.012	70	70	143.90	143.30	0.30
14	0	0	0	0.012	70	70	144.13	143.30	0.83
15	0	0	0	0.012	70	70	143.33	143.30	0.03
16	0	0	0	0.012	70	70	142.67	143.30	–0.63
17	0	0	0	0.012	70	70	142.47	143.30	–0.83

a Note: *x*_1_ and *X*_1_—biomass weight/extraction solvent ratio, *x*_2_ and *X*_2_—extraction temperature [°C], and *x*_3_ and *X*_3_—extraction time [min].

**Table 3:** Results of ANOVA Analysis for Quadratic Model Regression of the TPC in the Aqueous Extract of Olive Tree Leaves^a^

interaction type	source	TPC [μg GAE/mL]					
		coefficient factor	SS	df	MS	*F*-value	*p*-value
	model	143.300	5750,334	9	638,926	119.874	<0.0001*
linear	*x*_1_	12.901	1331.538	1	1331.538	2493.984	<0.0001*
	*x*_2_	18.705	2799.016	1	2799.016	5242.585	<0.0001*
	*x*_3_	8.721	608.482	1	608.482	1139.692	<0.0001*
quadratic	*x*_1_^2^	–4.195	74.097	1	74.097	138.784	0.0003*
	*x*_2_^2^	–12.088	615.190	1	615.190	1152.257	<0.0001*
	*x*_3_^2^	–7.340	226.845	1	226.845	424.882	<0.0001*
interaction	*x*_1_*x*_2_	–0.453	0.819	1	0.819	1.534	0.283
	*x*_1_*x*_3_	–1.250	6.250	1	6.250	11.706	0.027*
	*x*_2_*x*_3_	–0.808	2.608	1	2.608	4.885	0.092
residual			37.31	7	5.330		
pure error			2.136	4	0.534		
LoF			35.171	3	11.724	21.959	0.006*
core total			5787.644	16			

a Note: SD = 19.02; mean = 132.18; *R*^2^ = 0.9936; *R*_adj_^2^ = 0.9853; *R*_pred_^2^ = 0.9948; coefficient of variation (CV) % = 14.3; adequate precision = 36.5057; PRESS = 30.2435; SS—sum of squares; MS—mean square; PRESS—predicted residual error sum of squares; df–degree of freedom; * significant for *p* < 0.05.

## 3 Discussion

The ultimate goal of the research presented in this paper was to establish an effective biosynthesis method for AgNPs utilizing aqueous olive leaf extract. The process followed a two-stage procedure, wherein optimization was conducted through single-factor experiments for each step, namely, the extraction of polyphenols and the synthesis of AgNPs, using carefully selected parameters.

### 3.1 Single-Factor Experiment for Olive Tree Leaf Extraction

**Figure 1:**
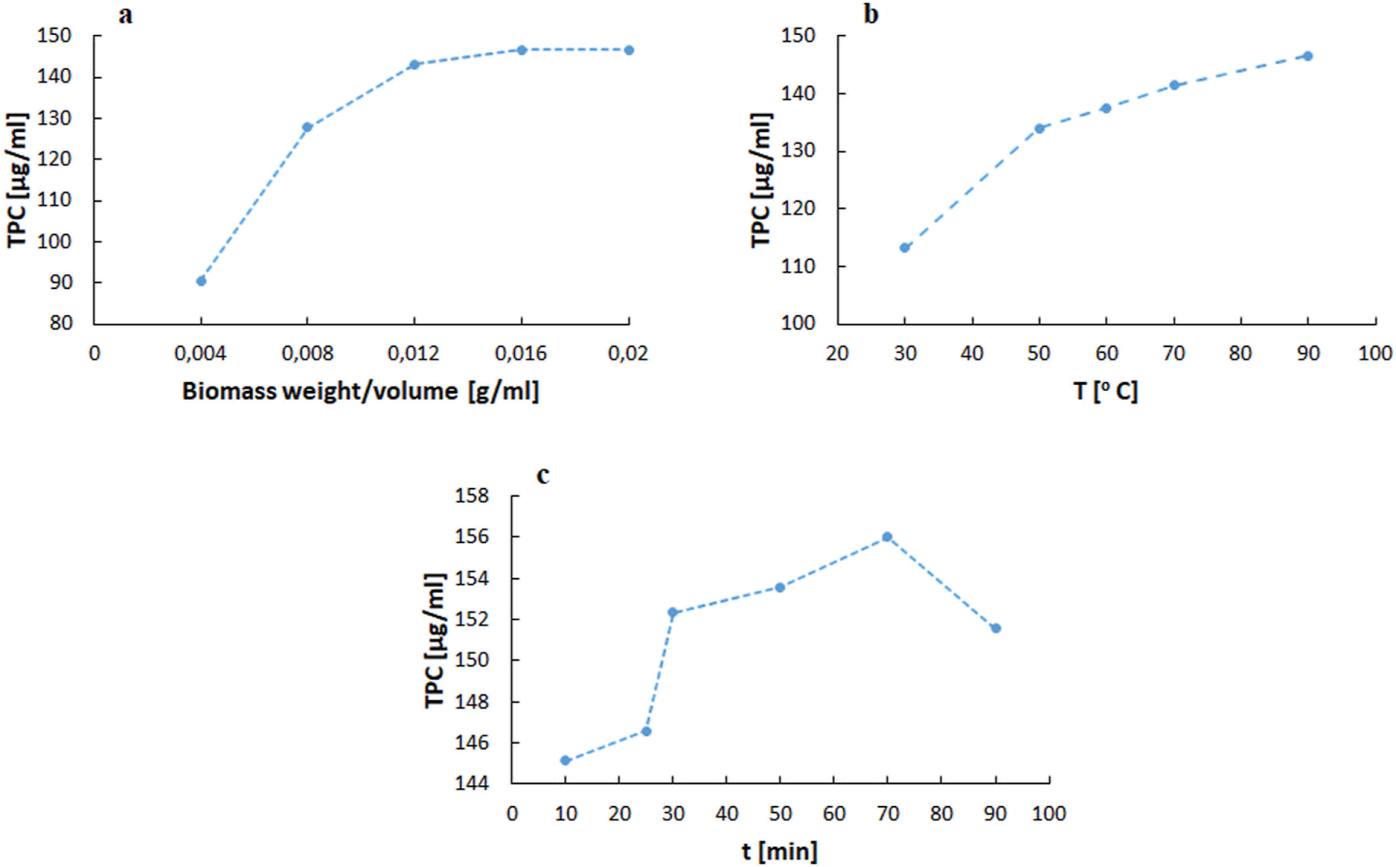
Effect of single parameters: (a) biomass to solvent volume ratio, (b) temperature, and (c) time on the TPC value [μg GAE/mL].

The study began with developing an optimization approach for the initial step of extracting polyphenolic compounds from olive tree L. Statistical calculations were performed using selected input values for the ratio of biomass to solvent volume, temperature, and extraction time (Figure 1).

The effect of the biomass/solvent ratio, ranging from 0.004 to 0.02 [g/mL], on the extraction efficiency, expressed as the total concentration of polyphenols (TPC), was assessed as the first parameter while keeping the other extraction conditions constant (25 min and 90 °C; Figure 1a). The results showed a steady increase in the polyphenols obtained in the aqueous extract, with a maximum TPC value of 143.67 [μg GAE/mL] observed for 1.6 g of olive leaves. When using 2 g of biomass, the amount of determined polyphenols in the aqueous extract is almost identical to that obtained with 1.6 g. The parameter values (−1) 0.8 g, (0) 1.2 g, and (1) 1.6 g were selected for statistical calculations.

The number of polyphenols in the extract increased with increasing temperature, while keeping other parameters constant at 0.016 [g/mL] and 25 min. The maximum yield was obtained at 90 °C, which was 146.57 [μg GAE/mL] (Figure 1b). To optimize the process, the temperature levels (−1) 50 °C, (0) 70 °C, and (1) 90 °C were selected. The maximum temperature that we used to extract olive leaves was 90 °C. There were mainly two reasons for this. First, we did not want to use a temperature equal to the boiling point of the solvent used for extraction, which in this case was water, because despite the plastic container being closed, we observed a loss in the solvent level during the process. This caused the filtration process of ground leaves and the determination of polyphenols using the Folin–Ciocalteau method to be imprecise compared to other experimental samples obtained at lower temperatures. Second, our concerns about the possible complete decomposition of plant cells or the formation of poorly soluble or water-insoluble forms of polyphenols and, therefore, their inefficient transfer from the cell wall to the solvent played a key role. This is often encountered when temperatures exceed 80 °C during the extraction of plant materials like leaves. This is undesirable from the point of view of efficient optimization; therefore, the most effective values of process parameters must be selected to find the maximum value of the independent variable, expressed here during extraction by the TPC value.

The last parameter tested was the extraction duration, which was tested as the last parameter in the range of 10–90 min (Figure 1c). The maximum polyphenol (TPC) concentration was obtained at 70 min, with a TPC value of 156 [μg GAE/mL]. A slight decrease in the TPC value was observed after 90 min. The final time values selected for the optimization model levels were (−1) 50 min, (0) 70 min, and (1) 90 min. Based on the single-factor experiments, coded values were assigned to the levels marked as (−1), (0), and (1) in the Box–Behnken statistical design. The matrix of the model comprised 17 experiments, including five central points, and is presented in Tables 1 and 2.

### 3.2 Optimization of the Extraction Process of Polyphenolic Compounds from Olive Tree L.

The data collected from the experimental TPC values obtained during olive leaf extraction, based on the Box–Behnken model matrix described in Table 2, enabled linear regression analysis using the surface response methodology. The statistical model utilized three independent variables: biomass/solvent volume ratio, temperature, and time, resulting in 17 experimental conditions. Table 2 presents the real and coded values of the input variables, the experimental- and model-predicted TPC values, and the residuals reflecting the difference between them.

Optimization aimed to determine the extraction conditions that would result in the highest value of TPC in μg GAE/mL. The analysis of experimental data revealed that the highest TPC value of 156 [μg GAE/mL] was obtained at a biomass/volume ratio of 0.016, a temperature of 90 °C, and an extraction time of 70 min. The average TPC value, calculated based on all extraction experiments, was 132.18 [μg GAE/mL]. A polynomial regression equation was developed using the Box–Behnken model used to predict the TPC value [μg GAE/mL] obtained from olive leaf extraction under various conditions, determined by the appropriate ratio of biomass/volume of solvent, temperature, and extraction period (eq 4)
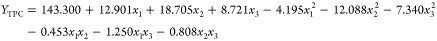
4where *x*_1_, *x*_2_, and *x*_3_ describe the coded independent variables: biomass/solvent volume ratio, temperature, and time, respectively. Figure S1 (see Supporting Information) illustrates the linear relationship between the TPC values [μg GAE/mL] obtained from the olive tree leaf extract experiment and the values predicted by the model based on the regression equation. The linearity and deviations from it provide information about the accuracy of the data match. The model and its final equation were verified by the ANOVA test, and the results are presented in Table 3.

The ANOVA test was conducted to confirm the model’s usefulness and reliability (Table 3). The *p*-value, *R*^2^, *R*_adj_^2^, *R*_pred_^2^, *F*-value of the model, LoF significance, PRESS, CV, and adequate precision parameters were all used to assess the model’s reliability and the optimal extraction conditions. The linear and quadratic coefficients obtained for the variables biomass/volume of solvent extraction ratio (*x*_1_), temperature (*x*_2_), and time (*x*_3_) were significant, whereas only the interaction coefficient between the biomass weight and solvent volume was significant for predicting the model. The other two coefficients concerning the interaction between the biomass/solvent volume and extraction time (*x*_1_*x*_3_) and between the temperature and time (*x*_2_*x*_3_) were found to be insignificant and were thus not included in the final regression equation (eq 5).
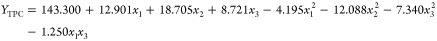
5

The Pareto graph (see Supporting Information: Figure S2) showed the effect of individual parameters and their interaction and is essential for optimizing the olive extraction efficiency, listing them in the order of greatest to least.

The *F*-value of the model was 18.015, and the low *p*-value of 0.003 indicated that that model was significant. The *R*^2^ and *R*_adj_^2^ coefficients had high values, exceeding 0.9 and amounting to 0.9936 and 0.9853, respectively, indicating that the model was well-characterized by 98.53% of all possible variances. The *R*_pred_^2^ coefficient, which was equal to 0.9948, was also satisfying. The value of the CV for the obtained olive leaf extraction model, equal to 14.3%, is lower than 20%, which usually allows the model to be recognized as acceptable and satisfactory because the variability of the obtained data is relatively low and the TPC values are very close to surrounding the mean value. Therefore, this model is stable and useful for determining the optimal conditions for the extraction process.

**Figure 2:**
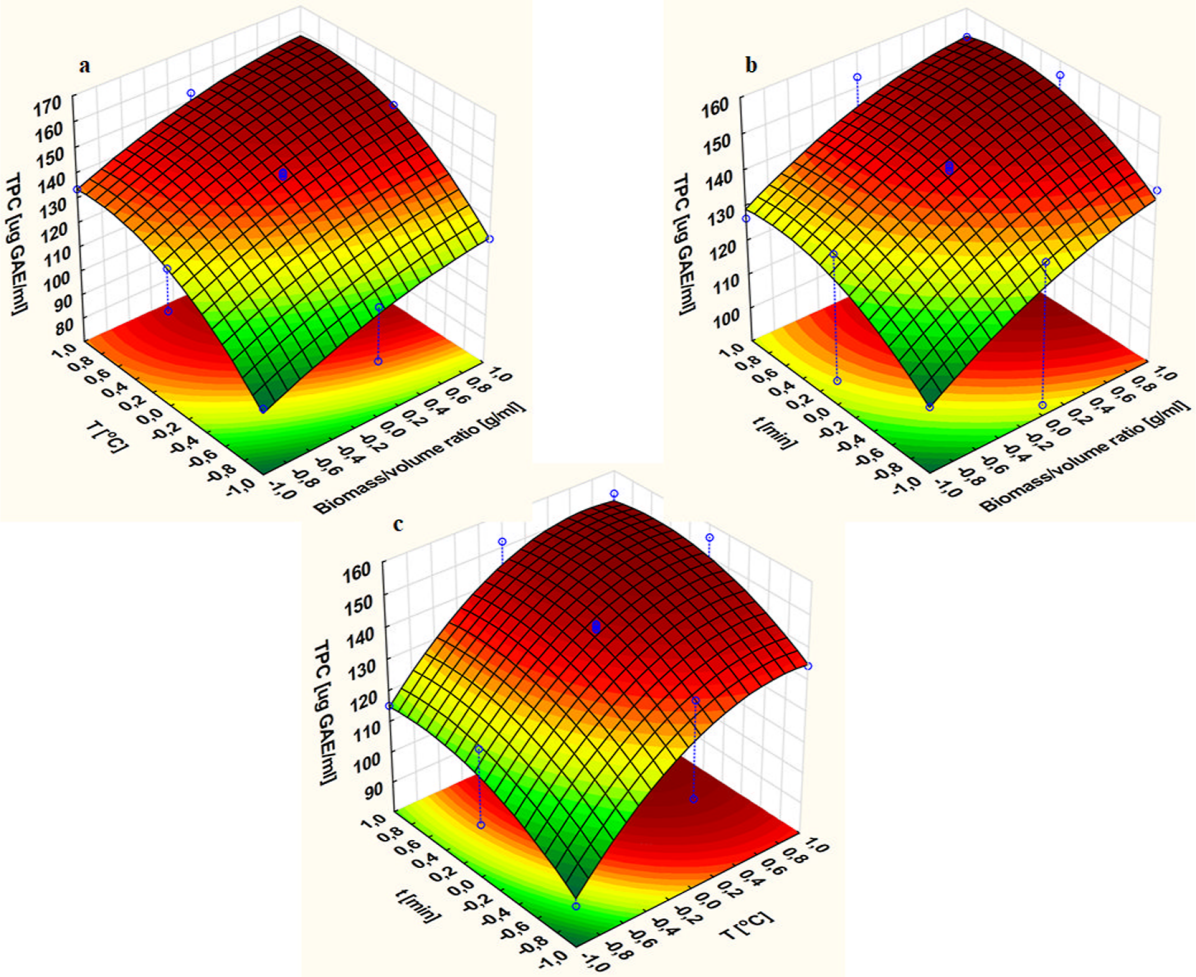
Response surface 3D plots for the result of an interaction effect between (a) biomass/volume ratio value vs temperature, (b) biomass/volume ratio vs time, and (c) temperature vs time on the TPC yield.

The calculated value of adequate precision was 36.5057, which exceeded 4 and indicated that the model can be used to precisely assess the optimal extraction conditions through analysis of the built three-dimensional surface response curves (Figure 2). The 3D response surface plots graphically depicted the existing correlation between the biomass/volume ratio value vs temperature (Figure 2a), biomass/volume ratio vs time (Figure 2b), and temperature vs time (Figure 2c) and determined their impact on the final extraction yield (TPC).

Figure 2a shows a three-dimensional response surface that illustrates the impact of two indicated parameters of the extraction process, such as the biomass/solvent ratio and temperature, on the value of its efficiency expressed as TPC extracted from a given amount of dried olive leaves. For the third independent variable of the process, time, a constant, middle value from the range selected for research was adopted. It should be noted that the indicated interaction is not significant for the process, which was confirmed by using the Pareto graph. On the other hand, the impact of both temperature and the biomass/solvent ratio is important for the extraction model in the case of linear and quadratic interaction. Finally, the temperature is indicated as the more significant value for the process in question. The interpretation allows for the indication that as the temperature increases, a large increase in the TPC value is observed, with the maximum located in the range of 70–90 °C. It should be noted that extraction efficiency also increases when the amount of biomass used is expanded with the optimum value at 1.6 g, which is in accordance with the result obtained during the single-factor experiment (Figure 1a).

The interaction between the biomass/solvent ratio and the duration of the extraction process while maintaining a constant temperature value, illustrated in the form of a three-dimensional response surface in Figure 2b, is the only one considered as significant according to the calculations of the obtained statistical model. Therefore, its analysis is crucial to determine optimal conditions for effective extraction. The interpretation of the submitted data indicates that both an increase in the biomass/solvent ratio and the extraction time lead to an increase in the TPC value. This means that under conditions of prolonged contact of the plant material with the solvent, the process is more efficient. The maximum extraction efficiency should be found based on the highest value of the biomass/solvent ratio and the time from the range between 75 and 85 min.

Figure 2c is a three-dimensional representation, insignificant for determining the optimal conditions of the interaction between the temperature and time of olive leaf extraction from the point of view of statistical calculations. The biomass/solvent ratio is constant with an intermediate value of 0.012. Each of these parameters is important in terms of the influence of the linear and quadratic effects; however, also in this case, temperature is more important. A higher total content of polyphenols is extracted from the leaves as the extraction temperature increases with a maximum located between 70 and 90 °C. The same correlation is also observed in the case of extraction time.

### 3.3 Validation of Optimal Model Conditions for Aqueous Extraction of Olive Tree L.

The comparative studies of the maximum TPC values predicted by the extraction model under optimal conditions of 0.016 biomass/solvent volume ratio, 80 °C temperature, and 80 min time and the corresponding experimental results performed confirm the final utility of the model.

The results show that the difference between the actual (160.67 μg/mL) and model-predicted TPC (159.81 μg/mL) values is very small (0.86), confirming the correctness of the model and suggesting its potential usefulness for determining the extraction efficiency of polyphenols from olive tree L., including in pilot trial experiments.

It should also be emphasized that the presented olive leaf extraction method was designed to follow the currently growing trend of optimizing each technological process. It is simple and easy to make and does not require the use of sophisticated and advanced laboratory equipment, which makes its cost low and its negative impact on the environment strongly reduced. The advantages offered by the above system give it a lead over most of the existing articles in the literature that focused on the extraction of bioactive substances from olive leaves.

First, only water was proposed as the extraction solvent. This differs from previous experiments, where researchers based work on the use of organic solvents, mainly 70–80% ethanol but also methanol or their mixtures with water.^11−14,16,20,24−27,29,31,33^ Water is an eco-friendly, safe, and green solvent, especially for food applications.

In addition, manuscripts related to olive leaf extraction mostly focus not on optimization issues but on confirming its antioxidant,^6,11,25^ antibacterial,^16,28^ or anticancer^25^ properties, with the aim of determining its usefulness in various industrial fields.

On the other hand, examples from the scientific literature that take into account extraction issues provide confirmation that temperature, time, or biomass/solvent ratio play a decidedly important role for this plant material. These parameters as independent variables have been used in studies conducted by the groups of Şahin and Şamli,^24^ Wang et al.,^25^ Giacometti et al.,^26^ or Oliveira et al.^12^

Another aspect that draws attention in the literature on olive leaf extraction is the use of technologically advanced extraction methodologies, especially including alternative energy sources: microwaves,^24,28,29^ ultrasound,^11,24−27^ or methods requiring the introduction of high pressure (PLE).^33^ These methods, despite shortening the extraction time, require the use of specific equipment that increases the cost of the process and energy consumption, which again puts our proposed extraction system in a favorable perspective.

Şahin and Şamli^24^ proposed sonochemical extraction of olive leaves using ethanol at a concentration of 50%, occurring optimally in 60 min. However, this required the use of an ultrasonic bath, followed by a centrifuge to separate the biomass from the solvent. The same method was used by Wang’s group,^25^ who performed the extraction at 50 °C for 50 min using methanol as the solvent. Extraction under ultrasonic conditions was also carried out by Japón-Luján’s group.^27^ The different organic solvents were compared, with the additional need to use an evaporator to evaporate the solvent and a stirring device. Taamalli et al.^31^ compared the extraction of olive leaves using microwaves, supercritical conditions, and high-pressure conditions with the traditional method. Extraction using the traditional method was much longer than other methods, but it did not require the use of additional equipment such as a device emitting microwaves, an oven, or pressure-controlling pumps.

### 3.4 Single-Factor Experiment for Olive Tree Leaf Extract Assisted in Biosynthesis of AgNPs

**Figure 3:**
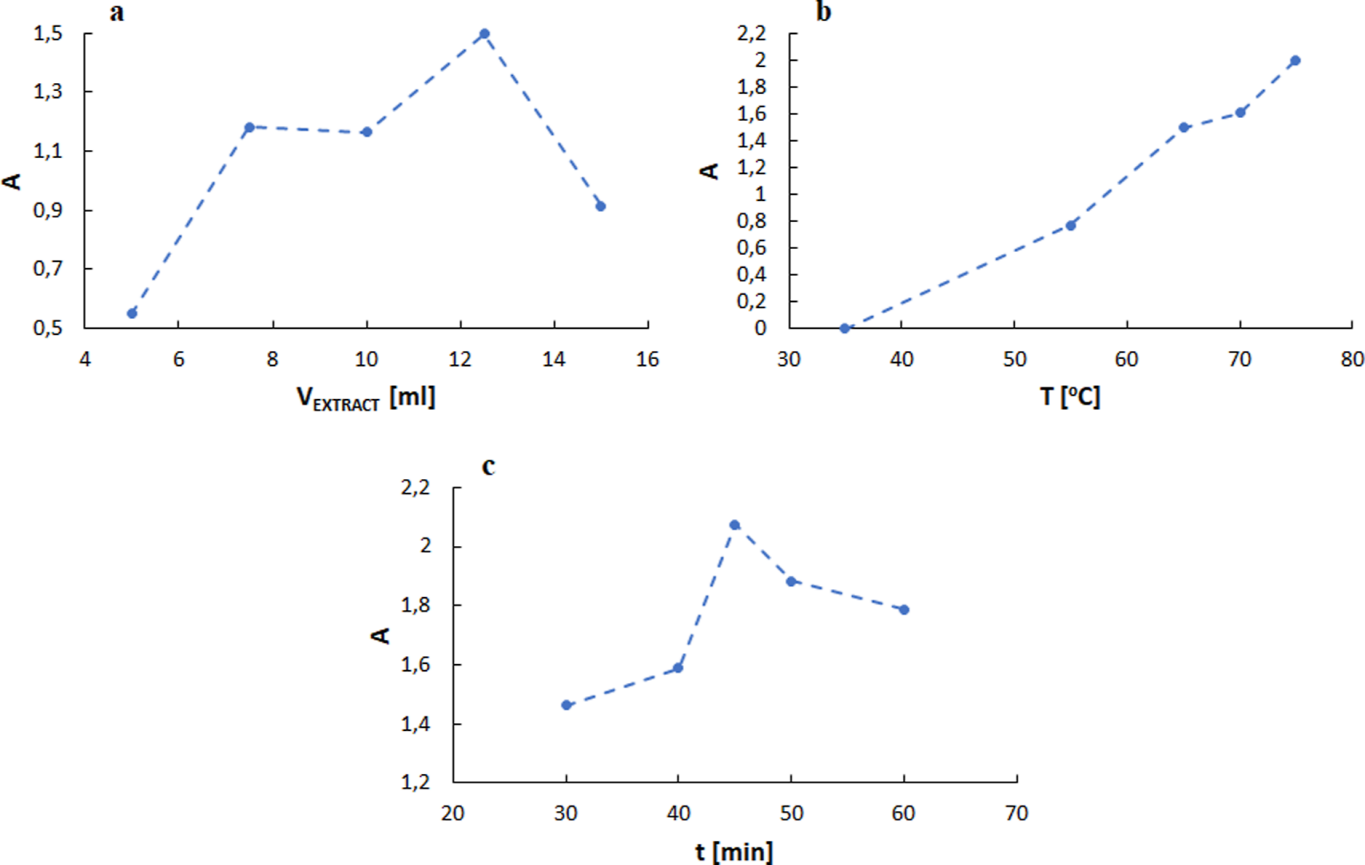
Effect of single parameters: (a) volume of the extract (mL), (b) temperature [°C], and (c) time [min] on the yield of biosynthesis of AgNPs.

The second part of the study focused on developing optimal conditions for the biosynthesis of AgNPs using an aqueous olive extract containing an optimized amount of polyphenols. The same strategy as in the extraction stage was adopted, with single-factor experiments conducted for selected parameters: extract volume, temperature, and biosynthesis time, leading to the determination of levels for the Box–Behnken factorial design matrix. The maximum absorbance value of the surface plasmon resonance peak (*A*) was taken as the yield of AgNP biosynthesis. Successive tests were carried out with extract volumes of 5, 7.5, 10, 12.5, and 15 mL, followed by syntheses at temperatures of 35, 55, 65, 70, and 75 °C, and finally, time selection was made within the range of 30–60 min (Figure 3).

Based on these results, the input values for the appropriate conditions for the biosynthesis of AgNPs were selected, which later served as uncoded levels of the mathematical model.

First, the effect of the extract volume on NP synthesis efficiency, expressed by the absorbance value (*A*) at which the characteristic absorption band of AgNPs reaches its maximum, was examined (Figure 3a). The maximum efficiency of AgNP biosynthesis was achieved when 12.5 mL of the olive extract was used while keeping the other process parameters constant at 65 °C and 45 min. The absorbance value was 1.496. Therefore, the mathematical model levels were (−1) 10 mL, (0) 12.5 mL, and (1) 15 mL.

The second parameter was the temperature of the process (Figure 3b). At a low temperature of 35 °C, no formation of AgNPs was observed after the end of the synthesis or the day after. However, the absorption band from the NPs was observed when the temperature was gradually increased between 55 and 75 °C, using a constant extract volume of 12.5 mL and a synthesis time of 45 min. The highest efficiency value (*A* = 1.998) was obtained at the highest temperature of 75 °C. We suppose that an increase in temperature could improve its efficiency. However, we decided to use a maximum temperature of 75 °C due to the aim of using the least demanding and harsh reaction conditions possible and reducing the risk of size changes or agglomeration of the resulting AgNPs at higher temperatures. Temperature values of 65, 70, and 75 °C were used as input levels (−1), (0), and (1) of the Box–Behnken plan, respectively.

Finally, a series of single-factor experiments were conducted for the duration of AgNP biosynthesis within 30–60 min (Figure 3c). The maximum efficiency of AgNP biosynthesis was achieved after 45 min at 75 °C with 12.5 mL of aqueous olive leaf extract, reaching an absorbance of 1.998. After a longer synthesis time, the maximum value of the absorption band gradually decreased. The input values of the independent variable time to the Box–Behnken model were 40, 45, and 50 min, coded as (−1), (0), and (1), respectively.

### 3.5 Optimization of Biosynthesis of AgNPs Using Aqueous Olive Tree L. Extract

The Box–Behnken mathematical model matrix for the NP biosynthesis process included 14 experiments performed in a specific order between the selected values of three independent variables, the volume of olive extract, temperature, and time as shown in Table 4. The table presents the appropriate uncoded and coded values of parameter levels and the absorbance values defining the biosynthesis efficiency of NPs obtained from both real experiments and those predicted by the model.

**Table 4:** Box–Behnken Factorial Design with the Independent Variables and Experimental and Predicted *A* Values as a Yield of Biosynthesis of AgNPs by Aqueous Olive Tree L. Extract^a^

run	coded variable			actual variable			TPC [mg GAE/g db]		
	*x*_1_ [mL]	*x*_2_ [°C]	*x*_3_ [min]	*X*_1_ [mL]	*X*_2_ [°C]	*X*_3_ [min]	actual	predicted	residual
1	–1	–1	0	10	65	45	1.165	1.195	–0.030
2	1	–1	0	15	65	45	0.916	0.989	–0.073
3	–1	1	0	10	75	45	1.682	1.609	0.073
4	1	1	0	15	75	45	1.860	1.830	0.030
5	–1	0	–1	10	70	40	1.220	1.254	–0.034
6	1	0	–1	15	70	40	1.184	1.175	0.009
7	–1	0	1	10	70	50	1.366	1.375	–0.009
8	1	0	1	15	70	50	1.503	1.469	0.034
9	0	–1	–1	12.5	65	40	1.175	1.111	0.064
10	0	1	–1	12.5	75	40	1.588	1.627	–0.039
11	0	–1	1	12.5	65	50	1.247	1.208	0.039
12	0	1	1	12.5	75	50	1.883	1.947	–0.064
13	0	0	0	12.5	70	45	1.603	1.596	0.007
14	0	0	0	12.5	70	45	1.588	1.596	–0.008

a Note: *x*_1_ and *X*_1_—extract volume [mL], *x*_2_ and *X*_2_—extraction temperature [°C], and *x*_3_ and *X*_3_—extraction time [min].

The aim was to find the conditions that would yield the maximum surface plasmon resonance absorbance value (*A*) for the band located at the wavelengths identifying their formation. The maximum value of the absorbance peak was observed when the AgNPs synthesis was carried out at 75 °C for 50 min using 12.5 mL of extract from dried olive leaves, according to the experimental values provided in the Box–Behnken plan. The average value of the absorbance band (*A*) obtained from all experimental data was 1.427.

The predicted values of the response, which is the efficiency of biosynthesis expressed as the maximum absorbance (*A*), were determined for each conducted experiment included in the Box–Behnken model based on linear regression analysis.

Using Statistica 13.3 software, linear regression analysis was performed, resulting in the second-order polynomial regression equation without considering the significance of the effects of the synthesis parameters, as shown below (eq 6)
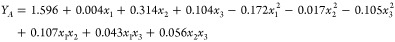
6where *x*_1_, *x*_2_, and *x*_3_ describe the coded value of input variables: extract volume, temperature, and time, respectively. Figure S3 (see the Supporting Information) shows a high level of agreement between experimental and statistical model data.

The assessment of the significance of the model and the individual effects included in the equation was based on the ANOVA test, and the results are presented in Table 5.

**Table 5:** Results of ANOVA Analysis for Quadratic Model Regression of the Yield of AgNP Synthesis by Aqueous Extract of Olive Tree L.^a^

interaction type	source	TPC [μg GAE/mL]					
		coefficient factor	SS	df	MS	*F*-value	*p*-value
	model	1.596	1.054	9	0.1171	18.015	0.003*
linear	*x*_1_	0.004	0.0001	1	0.0001	1.000	0.500
	*x*_2_	0.314	0.788	1	0.788	7000.111	0.008*
	*x*_3_	0.104	0.087	1	0.087	769.138	0.023*
quadratic	*x*_1_^2^	–0.172	0.095	1	0.095	845.174	0.022*
	*x*_2_^2^	–0.017	0.0009	1	0.0009	8.587	0.209
	*x*_3_^2^	–0.105	0.035	1	0.035	312.854	0.036*
interaction	*x*_1_*x*_2_	0.107	0.046	1	0.046	405.176	0.032*
	*x*_1_*x*_3_	0.043	0.007	1	0.007	66.509	0.078
	*x*_2_*x*_3_	0.056	0.012	1	0.012	110.509	0.060
residual			0.026	4	0.0065		
pure error			0.0001	1	0.0001		
LoF			0.0261	3	0.0087	77.382	0.083
core total			1.080	13			

a Note: SD = 0.29; mean 1.427; *R*^2^ = 0.9757; *R*_adj_^2^ = 0.9211; *R*_pred_^2^ = 0.9759; CV % = 20.3%; adequate precision = 14.2814; PRESS = 0.026; SS—sum of squares; MS—mean square; PRESS—predicted residual error sum of squares; df—degree of freedom; * significant for *p* < 0.05.

When the given *p*-value of effect coefficient did not exceed the limit value of 0.05, it was considered significant and included in the model. As a result, the final version of the polynomial regression equation (eq 7) included significant effect coefficients such as the linear effect of temperature (*x*_2_) and time (*x*_3_) and the quadratic effect of extract volume (*x*_1_^2^) and time (*x*_3_^2^), as well as the effect of interaction between extract volume and temperature (*x*_1_*x*_2_).
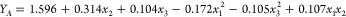
7

This was further summarized by the Pareto chart (see the Supporting Information: Figure S4). The values obtained for the regression coefficient (*R*^2^), adjusted regression coefficient (*R*_adj_^2^), and *R*_pred_^2^ were evaluated as a priority for the usefulness and quality of the model. Their values were higher than 0.9 and amounted to 0.9757, 0.9211, and 0.9759, respectively, suggesting that the built model has a high degree of accuracy and that 92.11% of model variances explain it well. In the next step, the model was characterized in terms of the significance of the linear and quadratic effect coefficients and the coefficient interpreting the interactions between the parameters of AgNPs biosynthesis by assessing the *p*-value.

**Figure 4:**
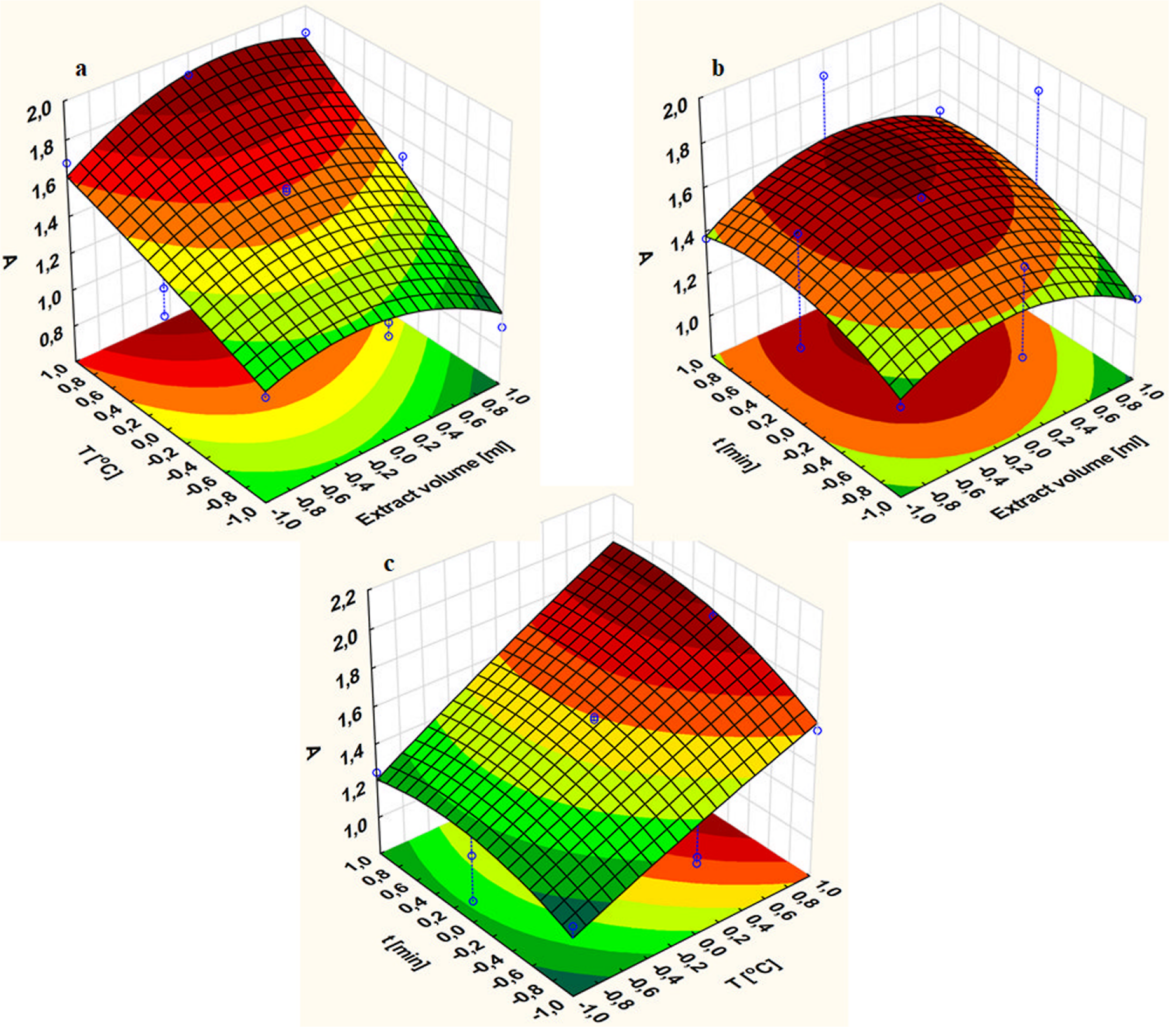
Response surface 3D plots for the result of an interaction effect between (a) extract volume vs temperature, (b) extract volume vs time, and (c) temperature vs time on the AgNPs biosynthesis yield.

The model’s *p*-value was assessed as significant with a value of 0.03 (<0.05), and the insignificance of the parameter called LoF confirmed the usefulness and precision of the proposed model. Other analyzed parameters included CV and adequate precision. The CV value, which was 20.3%, was higher than that achieved in the case of the extraction process but still fell within the range, allowing us to consider the model useful. The adequate precision, which was 14.2814, was higher than 4, meaning that we can generate and analyze 3D response surface plots (Figure 4) to determine the optimal conditions for AgNPs biosynthesis.

These plots represent the effect of interaction between the following parameters: extract volume vs temperature (Figure 4a), extract volume vs time (Figure 4b), and temperature vs time (Figure 4c) on the maximum absorbance value as a response.

### 3.6 Validation of Optimal Model Conditions for the Biosynthesis of AgNPs by Olive Tree L. Extract

Optimization results suggest that the most efficient synthesis of AgNPs [absorbance value (*A*)] using an aqueous olive tree leaf extract can be obtained under the statistically optimal conditions of 11 mL extract volume, 75 °C temperature, and 50 min synthesis time.

The obtained experimental result (1.955) confirms the accuracy of the predicted value (1.983), thus validating the model’s utility for evaluating the efficiency of AgNPs biosynthesis. The high similarity between the predicted and actual values is evident, with a difference of only 0.028.

### 3.7 Characterization of AgNPs Biosynthesized Using Olive Tree L. Extract

**Figure 5:**
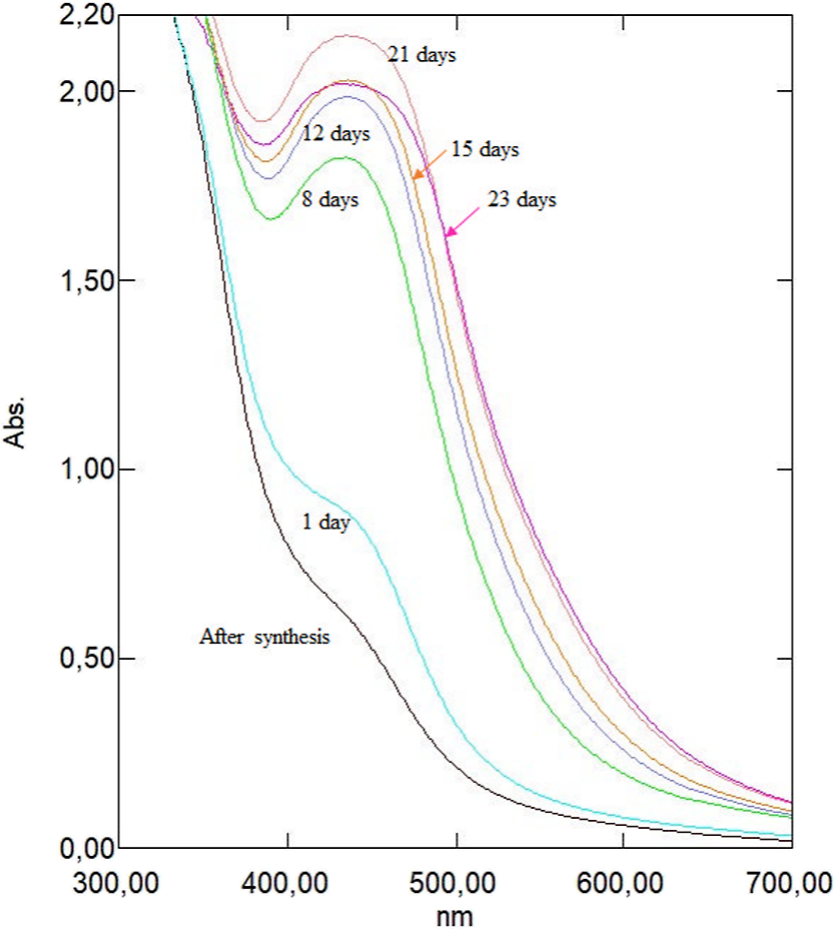
UV–vis spectrum monitoring of AgNPs’ stability after synthesizing 15.0 mL of olive tree L. extract.

During the bioreduction of AgNO_3_ with the optimized aqueous extract of olive tree leaves, a dark-yellowish-brown suspension was obtained, which indicated the formation of AgNPs. This was further confirmed by the recorded UV–vis spectra, where the maximum surface plasmon resonance absorbance appeared in the range characteristic for AgNPs at a wavelength between 420 and 443 nm (Figure 5).^45−48^

The absorption spectrum measured for the sample after biosynthesis confirms the presence of a low-intensity band in the wavelength range typical of the formation of AgNPs. However, completing the bioreduction process at a given temperature and placing the reaction mixture in a dark glass bottle stored at low temperature do not necessarily involve stopping the reduction reaction. Unreacted Ag^+^ ions may still be present in the solution and be reduced by polyphenols because they remain in constant contact in the postreaction mixture despite the low temperature. This effect can be seen as an increase in the intensity of the AgNP absorption band determining the efficiency of biosynthesis, which generally becomes sharper at the maximum point and changes in its shape. But it should be remembered that the rate of bioreduction may vary throughout the intended time range of sample storage and stability tests. This is evidenced precisely by the absorption bands recorded after 1, 8, 12, 15, and 21 days from biosynthesis, where its intensity increased and it became more shaped.

**Figure 6:**
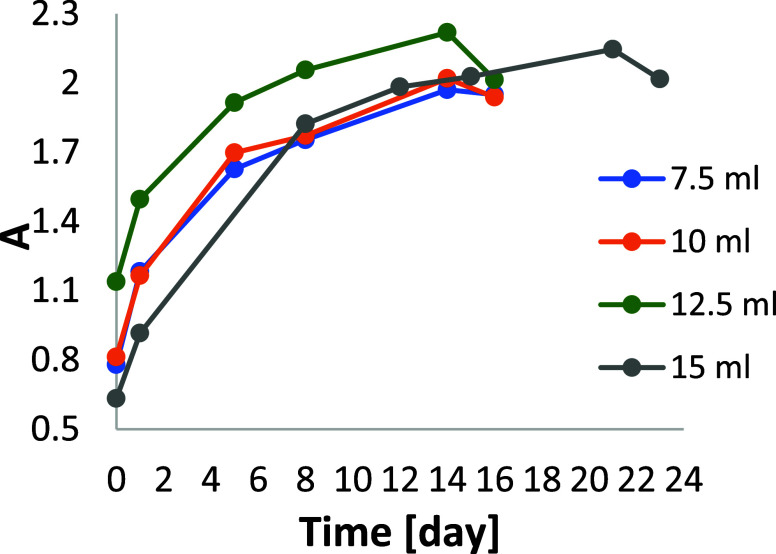
Stability test of AgNPs during storage time biosynthesized using 7.5, 10, 12.5, and 15 mL of olive tree L. extract.

To test the stability of the synthesized AgNPs, the height of the maximum absorption band was monitored using the UV–vis method for four samples synthesized with different volumes of olive leaf extract (7.5, 10, 12.5, and 15 mL) at various storage intervals (Figure 6). The result showed that only the sample synthesized with 15 mL of extract has high stability for 3 weeks, followed by a decrease in the absorption band. For the remaining samples, suspension stability was maintained for 2 weeks, possibly due to the greater amount of organic molecules on the AgNP surface. After a 2-week storage period for the postreduction samples, whose preparation required 7.5, 10, and 12.5 mL, respectively, no precipitate of AgNPs was observed but only a deepening of the intensity of the color of the test mixture. This may be due to the fact that the average diameter size of AgNPs for each sample increased but did not exceed 55 nm.

For each sample, an increase in the average diameter value was obtained in the measurement period between the first and second week. AgNPs with the largest average diameter were obtained by using 12.5 mL of extract for bioreduction. After 1 week, its value was approximately 46.5 nm, while after 2 weeks, it increased to 53.6 nm. The visual effect of this is a reduction in the intensity of the absorption band maximum in the electron spectrum.

**Figure 7:**
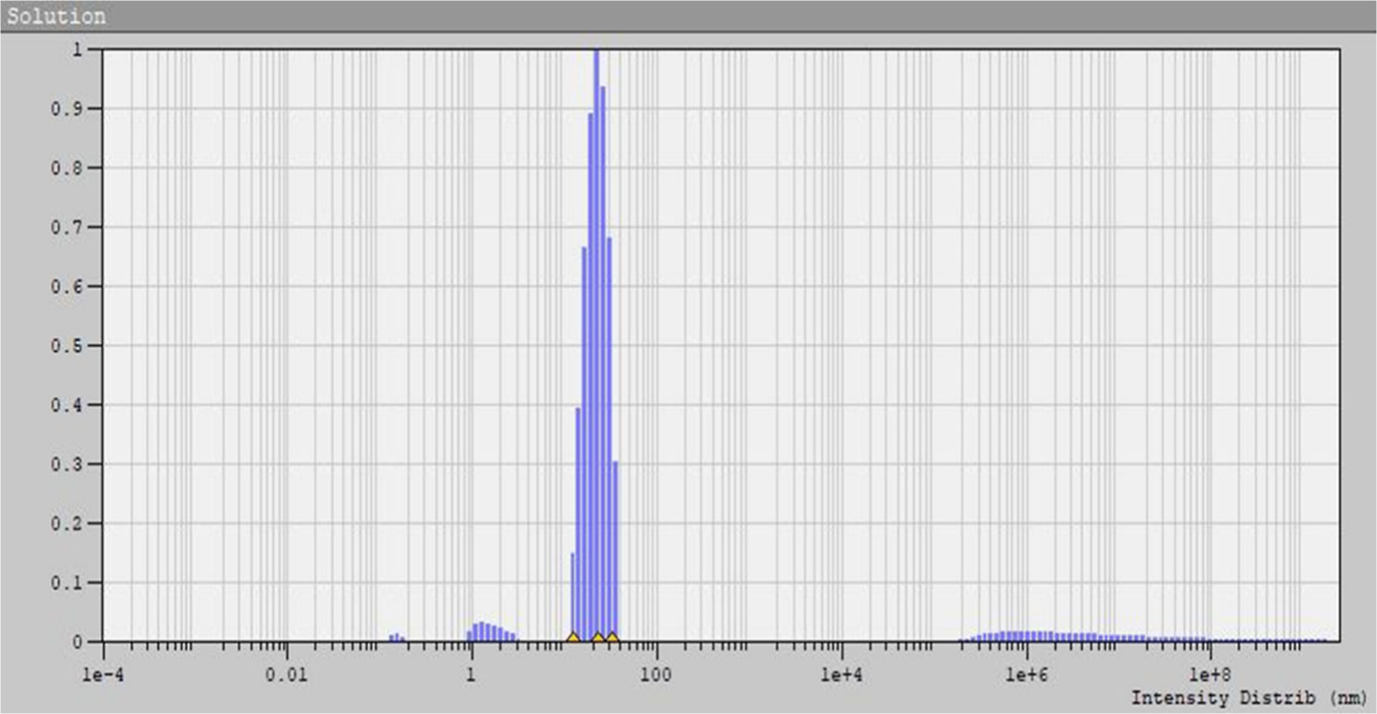
Size distribution of AgNPs after biosynthesis using olive tree L. extract under optimal conditions.

The synthesized AgNPs under optimal conditions were characterized using not only DLS but also the TEM method. The DLS measurement determined the radius of the NPs and their percentage composition in terms of size was obtained (Table 6 and Figure 7), revealing an average diameter of about 43 nm.

**Table 6:** Size Distribution of AgNPs after Biosynthesis under the Optimal Conditions

radius of NPs [nm]	percentage composition [%]	mean size [nm]
0.145	<1%	21.53
1.499	>3%	
21.56	>90%	

**Figure 8:**
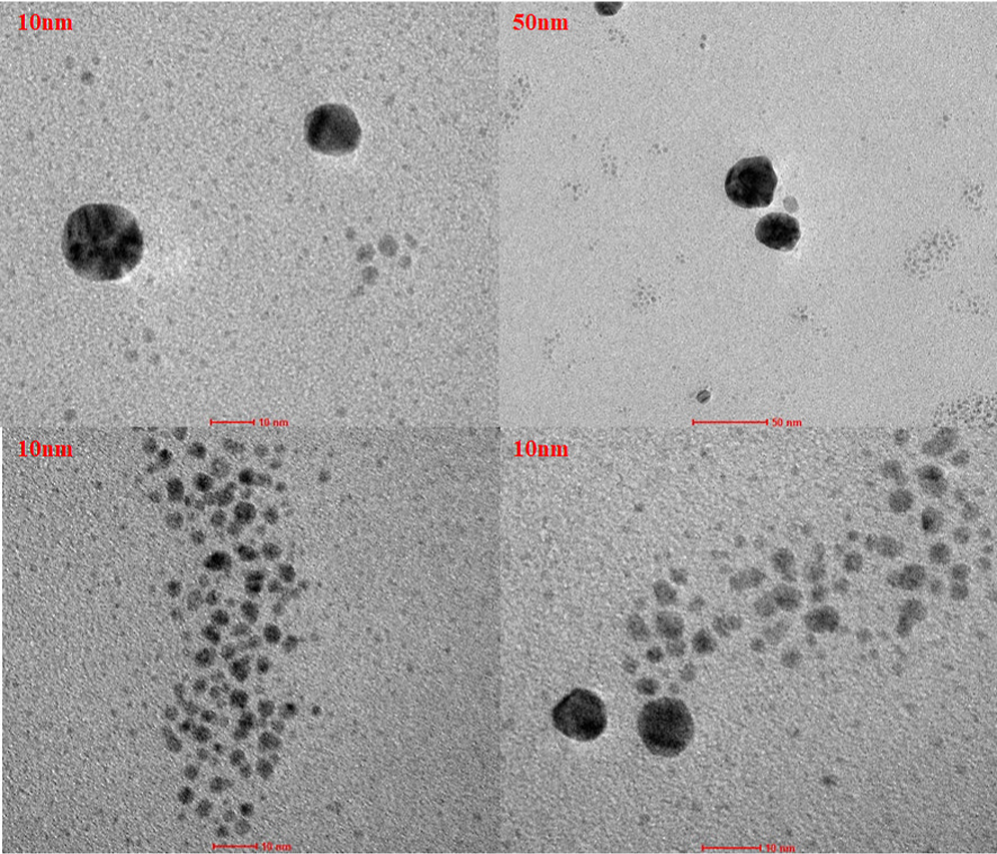
TEM images for AgNPs after biosynthesis using olive tree L. extract under optimal conditions.

Analysis of morphology of biosynthesized AgNPs with TEM shows their spherical shape without any tendency toward agglomeration (Figure 8). The small size and similar shape of AgNPs were also observed in case of examples found in the literature.^49^

**Figure 9:**
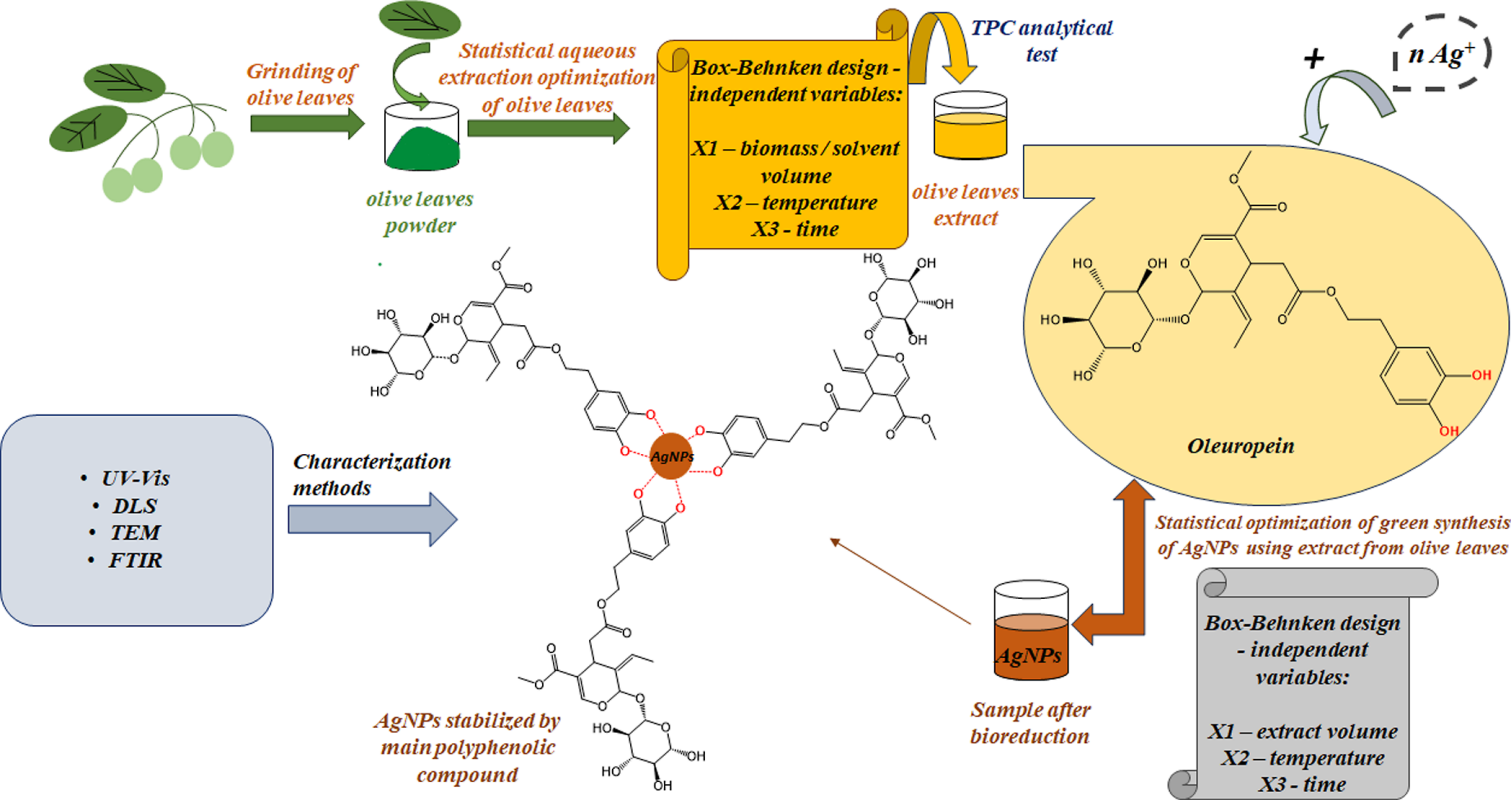
General scheme of the probable course of AgNPs formation and stabilization, based on the structure of oleuropein, the main component of the aqueous extract of olive leaves.

As mentioned earlier, olive leaves should definitely be classified as a group of plant sources rich in compounds with great potential of bioactivity.^50^ Commonly presented in the literature, the profile of substances confirmed to be present in the leaves of this plant abounds in polyphenolic compounds, terpenic acids, and sugars, the amounts of which oscillate depending on the conditions prevailing during cultivation.^34,51−53^ Among polyphenolic compounds, oleuropein is observed to be dominant, mostly accounting for 88–94% of their total amount. Therefore, it is assigned a responsible role as a natural reductant, an agent that stabilizes the formed AgNPs and, at the same time, protects them from the agglomeration process. For confirmation of the presence of phenolic groups in the optimized olive leaf extract, their participation in the biosynthesis process, and ultimately role in the stabilization of AgNPs, an analysis of functional groups and changes in their position in the FTIR spectra for the extract and biosynthesized NPs was performed (see Supporting Information: Figures S5 and S6). Phenolic groups, which may belong to polyphenolic compounds or aromatic alcohols, were observed in the form of broad peaks present in both FTIR spectra at wavelengths of 3430 cm^–1^ for the extract and 3435 cm^–1^ for the biosynthesized sample of AgNPs, respectively. For both samples, an absorption band corresponding to the occurrence of C–H functional groups at 2080 and 2088 cm^–1^ was also observed. In the FTIR spectrum of olive extract and biosynthesized nanosilver, a slight shift was observed for the band located at around 1630 cm^–1^, attributed to the stretching vibrations of the C=O groups that occur in the structure of aromatic ketones and carboxylic acids. In the sample obtained after the synthesis of AgNPs with the help of olive leaf extract, the appearance of a band of low intensity at a wavelength above 1000 cm^–1^ was observed, which is attributed to the presence of C–O bonds. Additionally, the presence in this sample of two small but broad peaks at a wavelength in the range of 520–600 cm^–1^ suggests the formation of a metallic form of silver. The type of bands and their shifts observed in the FTIR spectra of plant extract before and after the biosynthesis of AgNPs are consistent with the previously described studies.^46,48,54^ The changes observed in the FTIR spectra for tested samples: extract and biosynthesized AgNPs indicate that polyphenolic compounds found in olive extract can interact with the surface of nanosilver, form bonds with it, and cover their surface to protect them from agglomeration.^55^ The possible process of extraction and bioreduction of Ag^+^ ions involving oleuropein contained in olive leaf extract is illustrated in the general scheme (Figure 9).

**Figure 10:**
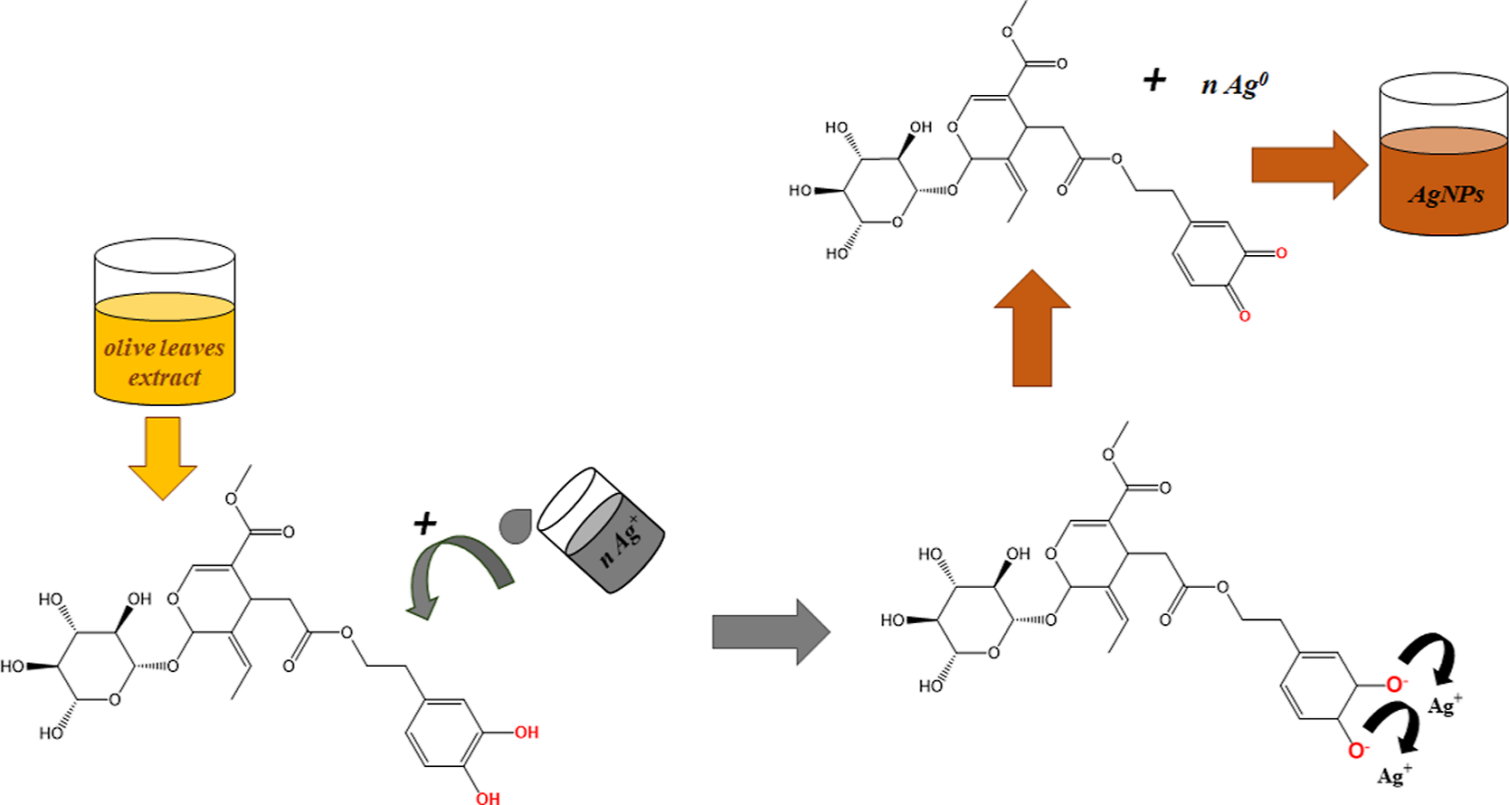
Possible predicted mechanism of bioreduction of the silver precursor (AgNO_3_) to AgNPs by oleuropein.

The formation of AgNPs with the polyphenolic compound—oleuropein takes place on the basis of a redox-type reaction.^55^ Hydroxyl groups located at the aromatic ring are key sites for the interaction of this biomolecule with silver ions. Their oxidation to a ketone functional group, occurring with the formation of a transition product: the enol form, is associated with the reduction of metal ions to the Ag(0) form due to electron transfer (Figure 10). The enol intermediate allows for the appearance of interaction of polyphenol with the surface of AgNPs.

## 4 Biosynthesis of AgNPs—Pilot Plant Design

The originality of this work focuses on the development of a guide for two important technological processes: olive leaf extraction and AgNP biosynthesis based on a mathematical optimization model, whose design is desirable from the point of view of the world’s and technology’s drive to develop green procedures with as little environmental impact as possible.

In the literature exist examples for preparation of a pilot plant in relation to plant leaf extraction processes.^56−60^ To the best of our knowledge, this is probably the first such article that discusses in detail the procedure for optimizing two interrelated process procedures under conventional conditions: extraction and biosynthesis of NPs using olive leaf extract on a small laboratory scale and finally the scaling up of it and the design of a pilot plant for the procedure of biosynthesis of AgNPs using prepared extract. The number of research articles focusing on pilot-scale olive leaf extraction is very limited. Additionally, available procedures are based on the use of an organic solvent.^57,61^ This is an extremely ecological, attractive, and economically viable approach. In the event of a change in the scalability of the process, it is possible to reduce the necessary experiments to a minimum while minimizing the consumption of reagents, energy, and time, compared to an approach where the search for optimal conditions would take place directly in the procedure on a larger scale.

**Figure 11:**
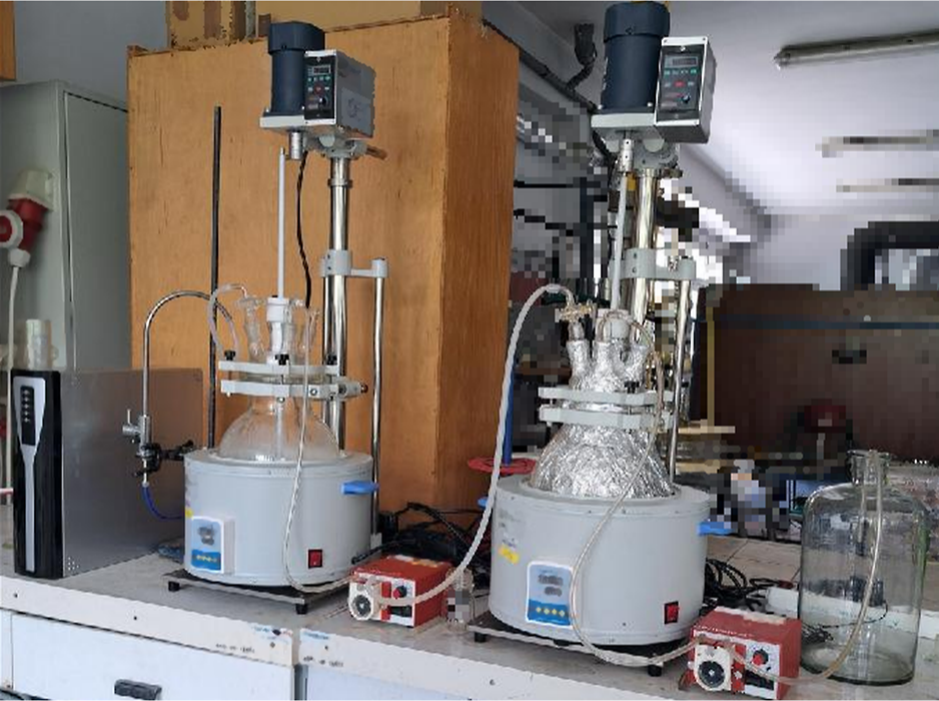
Pilot plant scaling up of biosynthesis of AgNPs using olive leaf extract.

The design of the pilot plant assumed the use of two reactors with a volume of 5 L. The media were transported from the extraction to the biosynthesis reactors due to the inclusion of peristaltic pumps in the reaction system (Figure 11).

The initial conditions for efficient extraction on a pilot scale were as follows: biomass/solvent ratio of 0.0016, 50 min as time of extraction, and temperature of extraction of 60 °C. The second stage was bioreduction of the AgNPs precursor: AgNO_3_ using a plant extract was carried out using two concentrations: 10^–3^ M and 5 × 10^–3^ M at 70 °C for 35 min, obtaining 59.81 and 31.37% efficiency, respectively.

It should be emphasized that the pilot experiments presented in this manuscript constitute only a diversification of the research, and they are not definitive. At the same time, they can be a preliminary strategy to create a final, well-functioning, and effectively functioning installation, where two processes of extraction and biosynthesis of AgNPs occur with high efficiency one after the other.

To achieve this goal, it is necessary to carry out further research related to the assessment of the amount of leaves or modification and determine the final values of the parameters for both processes on a larger scale. The authors are continuing their research in this area to develop the most effective synthetic process for scaling up, which will probably be presented in a separate paper.

## 5 Conclusions and Future Perspective

Climate changes, which are increasingly visible and tangible, and the related problems that people around the world struggle with to varying degrees put pressure on changes in the functioning of society. This is related to the development of all areas of life in accordance with the “zero waste” trend and the reduction of the negative impact on the environment in every possible aspect. These difficulties also reach the scientific world, where researchers are faced with the challenge of developing green synthetic protocols that will meet the Principles of Green Chemistry and the requirements of sustainable development. This applies to both research conducted on a laboratory scale and experiments prepared to be scaled up for industrial implementation. At the same time, an additional problem is connected with obtaining specific properties, the desired stability, and durability of the final material.

This is particularly important in the case of scientific fields that work at the nanoscale, such as nanotechnology, nanomedicine, or nanobiotechnology, among others. Designing nanomaterials with specific properties and applications according to the green protocol is very difficult because of the low stability and durability of the physicochemical properties of metal NPs as the basic element creating the final nanomaterial.

Another important aspect of the biosynthesis of NPs using extracts obtained from plants is paying attention to the time and economics of the process, especially from the point of view of industrial applications. In this case, future research should be based on the use of statistical models to reduce the number of experiments needed to obtain optimal conditions to a minimum by analyzing the most important parameters affecting the synthesis instead of each one separately.

**Figure 12:**
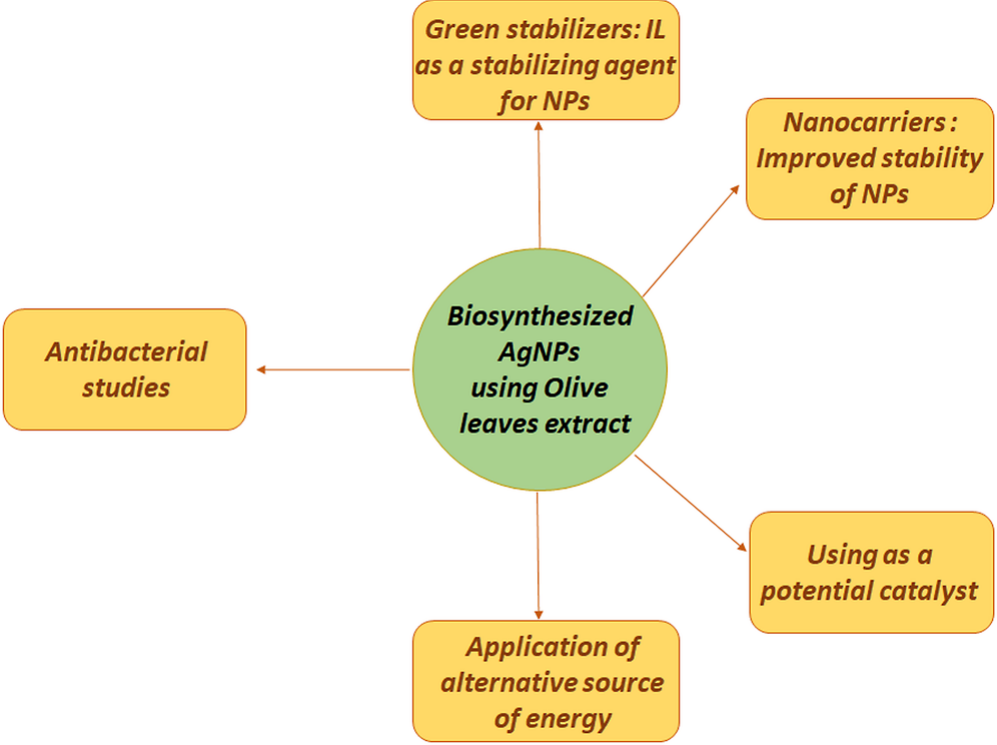
Future recommendation of the study path for AgNPs biosynthesized using olive tree leaf extract.

Based on the current literature, we prepared this article as an attempt to respond to the challenges and difficulties mentioned above. Its aim is to present a way to obtain a complex and green guide for the biosynthesis of AgNPs using an aqueous extract of olive leaves, starting from the development of optimal conditions for leaf extraction through optimizing the biosynthesis of AgNPs using statistical models in both cases, to finally lead to the design and creation of a pilot line on a larger scale. Additionally, the advantages of the presented NP synthesis methodology are low toxicity, low cost, and low energy consumption, which increases its profitability and reduces the negative impact on the environment and human health. Additionally, it was decided to raise the question of recommended paths for further applications of an optimized AgNPs system (Figure 12).

First of all, attention should be paid to the size and stability of AgNPs synthesized using olive leaf extract. They have a small average diameter not exceeding 50 nm and do not show symptoms of an agglomeration existence. The longest period of their stability is 3 weeks. Therefore, subsequent studies should focus on the use of a green stabilizing agent in the form of a surfactant or an ionic liquid enriched with a natural component in the structure. Here, several research aspects can be considered: statistical optimization of the synthesis of AgNPs in the presence of a stabilizer, examination of the impact of the stabilizer on the synthesis of AgNPs under conditions previously optimized for the system without stabilization, and assessment of the possible functions performed by the stabilizer.

The existence of interaction between AgNPs and polyphenolic compounds from olive leaf extract, confirmed by FTIR tests, encourages the use of additional nanocarriers in the system. This would make it possible not only to increase stability but also to consider the possibility of creating a new nanomaterial with specific properties and also to examine the mechanism of its formation by analyzing possible interactions between NPs, compounds derived from the extract, the stabilizer in the form of an ionic liquid, and the carrier.

An interesting aspect would also be a comparison of synthesis carried out under standard conditions with the type of experiment using alternative energy sources, also based on a statistical model. This is due to the shorter preparation times of various nanosystems observed in the literature in the presence of plant bioreducers with the simultaneous use of slightly milder reaction conditions.

Due to the presence of AgNPs, whose antibacterial properties have been widely known, it should be considered to check the effect of the performed nanosystems on various strains of bacteria.

Numerous literature studies confirm that this type of system can also be successfully used as a potential catalyst for various organic syntheses, including photocatalytic decomposition of organic dyes. In this case, it is also important to consider future research directions.

## Supporting Information Available

Linear relationship between the actual and predicted value of TPC as a result of extraction of olive tree L., Pareto graph of the standardized effect of independent variables and interaction between them for the aqueous extraction of olive tree L., linear relationship between the actual and predicted value of *A* as a response to the biosynthesis of AgNPs by olive tree L. extract, Pareto graph of the standardized effect of independent variables and interaction between them for the yield of biosynthesis of AgNPs, FTIR spectra for aqueous *O. europaea* L. extract, and FTIR spectra for sample after biosynthesis of AgNPs using aqueous *O. europaea* L. extract (PDF)

## References

[ref1] Ettadili F. E.; Aghris S.; Laghrib F.; Farahi A.; Saqrane S.; Bakasse M.; Lahrich S.; El Mhammedi M. A. Recent Advances in the Nanoparticles Synthesis Using Plant Extract: Applications and Future Recommendations. J. Mol. Struct. 2022, 1248, 131538 10.1016/j.molstruc.2021.131538.

[ref2] Vanlalveni C.; Lallianrawna S.; Biswas A.; Selvaraj M.; Changmai B.; Rokhum S. L. Green Synthesis of Silver Nanoparticles Using Plant Extracts and Their Antimicrobial Activities: A Review of Recent Literature. RSC Adv. 2021, 11 (5), 2804–2837. 10.1039/D0RA09941D. 35424248 PMC8694026

[ref3] Jadoun S.; Arif R.; Jangid N. K.; Meena R. K. Green Synthesis of Nanoparticles Using Plant Extracts: A Review. Environ. Chem. Lett. 2021, 19 (1), 355–374. 10.1007/s10311-020-01074-x.

[ref4] Rodríguez Sousa A. A.; Barandica J. M.; Aguilera P. A.; Rescia A. J. Examining Potential Environmental Consequences of Climate Change and Other Driving Forces on the Sustainability of Spanish Olive Groves under a Socio-Ecological Approach. Agriculture 2020, 10 (11), 509 10.3390/agriculture10110509.

[ref5] Hanbury D. On the Febrifuge Properties of the Olive (Olea europea, L.). Pharm. J. Prov. Trans. 1854, 353–354.

[ref6] Sifaoui I.; López-Arencibia A.; Martín-Navarro C. M.; Chammem N.; Reyes-Batlle M.; Mejri M.; Lorenzo-Morales J.; Abderabba M.; Piñero J. E. Activity of Olive Leaf Extracts against the Promastigote Stage of Leishmania Species and Their Correlation with the Antioxidant Activity. Exp. Parasitol. 2014, 141 (1), 106–111. 10.1016/j.exppara.2014.03.002. 24662269

[ref7] Clodoveo M. L.; Crupi P.; Annunziato A.; Corbo F. Innovative Extraction Technologies for Development of Functional Ingredients Based on Polyphenols from Olive Leaves. Foods 2021, 11 (1), 103 10.3390/foods11010103. 35010227 PMC8750173

[ref8] Fu S.; Arráez-Roman D.; Segura-Carretero A.; Menéndez J. A.; Menéndez-Gutiérrez M. P.; Micol V.; Fernández-Gutiérrez A. Qualitative Screening of Phenolic Compounds in Olive Leaf Extracts by Hyphenated Liquid Chromatography and Preliminary Evaluation of Cytotoxic Activity against Human Breast Cancer Cells. Anal. Bioanal. Chem. 2010, 397 (2), 643–654. 10.1007/s00216-010-3604-0. 20238105

[ref9] Bianco A.; Uccella N. Biophenolic Components of Olives. Food Res. Int. 2000, 33 (6), 475–485. 10.1016/S0963-9969(00)00072-7.

[ref10] Farag R. S.; El-Baroty G. S.; Basuny A. M. Safety Evaluation of Olive Phenolic Compounds as Natural Antioxidants. Int. J. Food Sci. Nutr. 2003, 54 (3), 159–174. 10.1080/0963748031000136306. 12775365

[ref11] Difonzo G.; Russo A.; Trani A.; Paradiso V. M.; Ranieri M.; Pasqualone A.; Summo C.; Tamma G.; Silletti R.; Caponio F. Green Extracts from Coratina Olive Cultivar Leaves: Antioxidant Characterization and Biological Activity. J. Funct. Foods 2017, 31, 63–70. 10.1016/j.jff.2017.01.039.

[ref12] Oliveira A. L. S.; Gondim S.; Gómez-García R.; Ribeiro T.; Pintado M. Olive Leaf Phenolic Extract from Two Portuguese Cultivars -Bioactivities for Potential Food and Cosmetic Application. J. Environ. Chem. Eng. 2021, 9 (5), 106175 10.1016/j.jece.2021.106175.

[ref13] Lee O. H.; Lee B. Y.; Lee J.; Lee H. B.; Son J. Y.; Park C. S.; Shetty K.; Kim Y. C. Assessment of Phenolics-Enriched Extract and Fractions of Olive Leaves and Their Antioxidant Activities. Bioresour. Technol. 2009, 100 (23), 6107–6113. 10.1016/j.biortech.2009.06.059. 19608415

[ref14] Liu Y.; McKeever L. C.; Suo Y.; Jin T. Z.; Malik N. S. A. Antimicrobial Activities of Olive Leaf Extract and Its Potential Use in Food Industry. ACS Symp. Ser. 2018, 1287, 119–132. 10.1021/bk-2018-1287.ch006.

[ref15] Agatonovic-Kustrin S.; Gegechkori V.; Morton D. W.; Tucci J.; Mohammed E. U. R.; Ku H. The Bioprofiling of Antibacterials in Olive Leaf Extracts via Thin Layer Chromatography-Effect Directed Analysis (TLC-EDA). J. Pharm. Biomed. Anal. 2022, 219, 114916 10.1016/j.jpba.2022.114916. 35809514

[ref16] Ahmed A. M.; Rabii N. S.; Garbaj A. M.; Abolghait S. K. Antibacterial Effect of Olive (Olea Europaea L.) Leaves Extract in Raw Peeled Undeveined Shrimp (Penaeus Semisulcatus). Int. J. Vet. Sci. Med. 2014, 2 (1), 53–56. 10.1016/j.ijvsm.2014.04.002.

[ref17] Martínez L.; Castillo J.; Ros G.; Nieto G. Antioxidant and Antimicrobial Activity of Rosemary, Pomegranate and Olive Extracts in Fish Patties. Antioxidants 2019, 8 (4), 86 10.3390/antiox8040086. 30987153 PMC6523725

[ref18] Barbaro B.; Toietta G.; Maggio R.; Arciello M.; Tarocchi M.; Galli A.; Balsano C. Effects of the Olive-Derived Polyphenol Oleuropein on Human Health. Int. J. Mol. Sci. 2014, 15 (10), 18508–18524. 10.3390/ijms151018508. 25318054 PMC4227229

[ref19] Umai D.; Vikranth A.; Meenambiga S. S. A Study on the Green Synthesis of Silver Nanoparticles from Olea Europaea and Its Activity against Oral Pathogens. Mater. Today: Proc. 2021, 44, 3647–3651. 10.1016/j.matpr.2020.10.681.

[ref20] Mylonaki S.; Kiassos E.; Makris D. P.; Kefalas P. Optimisation of the Extraction of Olive (Olea Europaea) Leaf Phenolics Using Water/Ethanol-Based Solvent Systems and Response Surface Methodology. Anal. Bioanal. Chem. 2008, 392, 977–985. 10.1007/s00216-008-2353-9. 18762919

[ref21] Alrugaibah M.; Yagiz Y.; Gu L. Novel Natural Deep Eutectic Solvents as Efficient Green Reagents to Extract Phenolic Compounds from Olive Leaves and Predictive Modelling by Artificial Neural Networking. Food Bioprod. Process. 2023, 138, 198–208. 10.1016/j.fbp.2023.02.006.

[ref22] Palma A.; Díaz M. J.; Ruiz-Montoya M.; Morales E.; Giráldez I. Ultrasound Extraction Optimization for Bioactive Molecules from Eucalyptus Globulus Leaves through Antioxidant Activity. Ultrason. Sonochem. 2021, 76, 105654 10.1016/j.ultsonch.2021.105654. 34198128 PMC8254034

[ref23] Chew S. Y.; Teoh S. Y.; Sim Y. Y.; Nyam K. L. Optimization of Ultrasonic Extraction Condition for Maximal Antioxidant, Antimicrobial, and Antityrosinase Activity from Hibiscus Cannabinus L. Leaves by Using the Single Factor Experiment. J. Appl. Res. Med. Aromat. Plants 2021, 25, 100321 10.1016/j.jarmap.2021.100321.

[ref24] Şahin S.; Şamli R. Optimization of Olive Leaf Extract Obtained by Ultrasound-Assisted Extraction with Response Surface Methodology. Ultrason. Sonochem. 2013, 20 (1), 595–602. 10.1016/j.ultsonch.2012.07.029. 22964032

[ref25] Wang B.; Qu J.; Luo S.; Feng S.; Li T.; Yuan M.; Huang Y.; Liao J.; Yang R.; Ding C. Optimization of Ultrasound-Assisted Extraction of Flavonoids from Olive (Olea Europaea) Leaves, and Evaluation of Their Antioxidant and Anticancer Activities. Molecules 2018, 23 (10), 2513 10.3390/molecules23102513. 30274358 PMC6222376

[ref26] Giacometti J.; Žauhar G.; Žuvić M. Optimization of Ultrasonic-Assisted Extraction of Major Phenolic Compounds from Olive Leaves (Olea Europaea L.) Using Response Surface Methodology. Foods 2018, 7 (9), 149 10.3390/foods7090149. 30200559 PMC6165173

[ref27] Japón-Luján R.; Luque-Rodríguez J. M.; Luque De Castro M. D. Dynamic Ultrasound-Assisted Extraction of Oleuropein and Related Biophenols from Olive Leaves. J. Chromatogr. A 2006, 1108 (1), 76–82. 10.1016/j.chroma.2005.12.106. 16442552

[ref28] Sahin S.; Samli R.; Tan A. S. B.; Barba F. J.; Chemat F.; Cravotto G.; Lorenzo J. M. Solvent-Free Microwave-Assisted Extraction of Polyphenols from Olive Tree Leaves: Antioxidant and Antimicrobial Properties. Molecules 2017, 22 (7), 1056 10.3390/molecules22071056. 28672807 PMC6152306

[ref29] Japón-Luján R.; Luque-Rodríguez J. M.; Luque De Castro M. D. Multivariate Optimisation of the Microwave-Assisted Extraction of Oleuropein and Related Biophenols from Olive Leaves. Anal. Bioanal. Chem. 2006, 385 (4), 753–759. 10.1007/s00216-006-0419-0. 16741775

[ref30] Beyecha Hundie K.; Aga Bullo T.; Mekonnen Bayisa Y.; Abdissa Akuma D.; Seid Bultum M. Optimization of Microwave-Assisted Hydro-Distillation Essential Oil Extracted from Rumex Crispus Leaves Using Definitive Screening Design. Arab. J. Chem. 2023, 16 (5), 104665 10.1016/j.arabjc.2023.104665.

[ref31] Taamalli A.; Arráez-Román D.; Barrajón-Catalán E.; Ruiz-Torres V.; Pérez-Sánchez A.; Herrero M.; Ibañez E.; Micol V.; Zarrouk M.; Segura-Carretero A.; Fernández-Gutiérrez A. Use of Advanced Techniques for the Extraction of Phenolic Compounds from Tunisian Olive Leaves: Phenolic Composition and Cytotoxicity against Human Breast Cancer Cells. Food Chem. Toxicol. 2012, 50 (6), 1817–1825. 10.1016/j.fct.2012.02.090. 22433985

[ref32] Taamalli A.; Arráez-Román D.; Ibañez E.; Zarrouk M.; Segura-Carretero A.; Fernández-Gutiérrez A. Optimization of Microwave-Assisted Extraction for the Characterization of Olive Leaf Phenolic Compounds by Using HPLC-ESI-TOF-MS/IT-MS2. J. Agric. Food Chem. 2012, 60 (3), 791–798. 10.1021/jf204233u. 22206342

[ref33] Okur I.; Namlı S.; Oztop M. H.; Alpas H. High-Pressure-Assisted Extraction of Phenolic Compounds from Olive Leaves: Optimization and Comparison with Conventional Extraction. ACS Food Sci. Technol. 2023, 3 (1), 161–169. 10.1021/acsfoodscitech.2c00346.

[ref34] Medina E.; Romero C.; García P.; Brenes M. Characterization of Bioactive Compounds in Commercial Olive Leaf Extracts, and Olive Leaves and Their Infusions. Food Funct. 2019, 10 (8), 4716–4724. 10.1039/C9FO00698B. 31304950

[ref35] Mittal A. K.; Chisti Y.; Banerjee U. C. Synthesis of Metallic Nanoparticles Using Plant Extracts. Biotechnol. Adv. 2013, 31 (2), 346–356. 10.1016/j.biotechadv.2013.01.003. 23318667

[ref36] Rashidipour M.; Heydari R. Biosynthesis of Silver Nanoparticles Using Extract of Olive Leaf: Synthesis and in Vitro Cytotoxic Effect on MCF-7 Cells. J. Nanostructure Chem. 2014, 4 (3), 112 10.1007/s40097-014-0112-3.

[ref37] Khalil M. M. H.; Ismail E. H.; El-Baghdady K. Z.; Mohamed D. Green Synthesis of Silver Nanoparticles Using Olive Leaf Extract and Its Antibacterial Activity. Arab. J. Chem. 2014, 7 (6), 1131–1139. 10.1016/j.arabjc.2013.04.007.

[ref38] Chang J.-S.; Lee Y.-P.; Wang R.-C. Optimization of Nanosized Silver Particle Synthesis via Experimental Design. Ind. Eng. Chem. Res. 2007, 46 (17), 5591–5599. 10.1021/ie061355f.

[ref39] Lalegani Z.; Seyyed Ebrahimi S. A. Optimization of Synthesis for Shape and Size Controlled Silver Nanoparticles Using Response Surface Methodology. Colloids Surfaces A Physicochem. Eng. Asp. 2020, 595, 124647 10.1016/j.colsurfa.2020.124647.

[ref40] Pourmortazavi S. M.; Taghdiri M.; Makari V.; Rahimi-Nasrabadi M. Procedure Optimization for Green Synthesis of Silver Nanoparticles by Aqueous Extract of Eucalyptus Oleosa. Spectrochim. Acta, Part A 2015, 136 (PC), 1249–1254. 10.1016/j.saa.2014.10.010. 25456666

[ref41] Hasnain M. S.; Javed M. N.; Alam M. S.; Rishishwar P.; Rishishwar S.; Ali S.; Nayak A. K.; Beg S. Purple Heart Plant Leaves Extract-Mediated Silver Nanoparticle Synthesis: Optimization by Box-Behnken Design. Mater. Sci. Eng., C 2019, 99, 1105–1114. 10.1016/j.msec.2019.02.061. 30889643

[ref42] Azmi S. N. H.; Al-Jassasi B. M. H.; Al-Sawafi H. M. S.; Al-Shukaili S. H. G.; Rahman N.; Nasir M. Optimization for Synthesis of Silver Nanoparticles through Response Surface Methodology Using Leaf Extract of Boswellia Sacra and Its Application in Antimicrobial Activity. Environ. Monit. Assess. 2021, 193 (8), 497 10.1007/s10661-021-09301-w. 34286386

[ref43] Everette J. D.; Bryant Q. M.; Green A. M.; Abbey Y. A.; Wangila G. W.; Walker R. B. Thorough Study of Reactivity of Various Compound Classes toward the Folin-Ciocalteu Reagent. J. Agric. Food Chem. 2010, 58 (14), 8139–8144. 10.1021/jf1005935. 20583841 PMC4075968

[ref44] Wirwis A.; Sadowski Z. Green Synthesis of Silver Nanoparticles: Optimizing Green Tea Leaf Extraction for Enhanced Physicochemical Properties. ACS Omega 2023, 8 (33), 30532–30549. 10.1021/acsomega.3c03775. 37636976 PMC10448680

[ref45] Felimban A. I.; Alharbi N. S.; Alsubhi N. S. Optimization, Characterization, and Anticancer Potential of Silver Nanoparticles Biosynthesized Using Olea Europaea. Int. J. Biomater. 2022, 2022, 1–12. 10.1155/2022/6859637. PMC9529486 36199851

[ref46] Sellami H.; Khan S. A.; Ahmad I.; Alarfaj A. A.; Hirad A. H.; Al-Sabri A. E. Green Synthesis of Silver Nanoparticles Using Olea Europaea Leaf Extract for Their Enhanced Antibacterial, Antioxidant, Cytotoxic and Biocompatibility Applications. Int. J. Mol. Sci. 2021, 22 (22), 12562 10.3390/ijms222212562. 34830442 PMC8621457

[ref47] De Matteis V.; Rizzello L.; Ingrosso C.; Liatsi-Douvitsa E.; De Giorgi M. L.; De Matteis G.; Rinaldi R. Cultivar-Dependent Anticancer and Antibacterial Properties of Silver Nanoparticles Synthesized Using Leaves of Different Olea Europaea Trees. Nanomaterials 2019, 9 (11), 1544 10.3390/nano9111544. 31671618 PMC6915347

[ref48] M. Awwad A.; M. Salem N.; O. Abdeen A. Biosynthesis of Silver Nanoparticles Using Olea Europaea Leaves Extract and Its Antibacterial Activity. Nanosci. Nanotechnol. 2013, 2 (6), 164–170. 10.5923/j.nn.20120206.03.

[ref49] Alowaiesh B. F.; Alhaithloul H. A. S.; Saad A. M.; Hassanin A. A. Green Biogenic of Silver Nanoparticles Using Polyphenolic Extract of Olive Leaf Wastes with Focus on Their Anticancer and Antimicrobial Activities. Plants 2023, 12 (6), 1410 10.3390/plants12061410. 36987100 PMC10057938

[ref50] Selim S.; Albqmi M.; Al-Sanea M. M.; Alnusaire T. S.; Almuhayawi M. S.; AbdElgawad H.; Al Jaouni S. K.; Elkelish A.; Hussein S.; Warrad M.; El-Saadony M. T. Valorizing the Usage of Olive Leaves, Bioactive Compounds, Biological Activities, and Food Applications: A Comprehensive Review. Front. Nutr. 2022, 9, 1008349 10.3389/fnut.2022.1008349. 36424930 PMC9678927

[ref51] Xie P.-J.; Huang L.-X.; Zhang C.-H.; Zhang Y.-L. lei. Phenolic Compositions, and Antioxidant Performance of Olive Leaf and Fruit (Olea Europaea L.) Extracts and Their Structure-Activity Relationships. J. Funct. Foods 2015, 16, 460–471. 10.1016/j.jff.2015.05.005.

[ref52] Medina E.; De Castro A.; Romero C.; Brenes M. Comparison of the Concentrations of Phenolic Compounds in Olive Oils and Other Plant Oils: Correlation with Antimicrobial Activity. J. Agric. Food Chem. 2006, 54 (14), 4954–4961. 10.1021/jf0602267. 16819902

[ref53] Hayes J. E.; Allen P.; Brunton N.; O’Grady M. N.; Kerry J. P. Phenolic Composition and in Vitro Antioxidant Capacity of Four Commercial Phytochemical Products: Olive Leaf Extract (Olea Europaea L.), Lutein, Sesamol and Ellagic Acid. Food Chem. 2011, 126 (3), 948–955. 10.1016/j.foodchem.2010.11.092.

[ref54] Bergal A.; Matar G. H.; Andac M. Olive and Green Tea Leaf Extracts Mediated Green Synthesis of Silver Nanoparticles (AgNPs): Comparison Investigation on Characterizations and Antibacterial Activity. Bionanoscience 2022, 12, 307–321. 10.1007/s12668-022-00958-2.

[ref55] Song Y.; Yang F.; Mu B.; Kang Y.; Hui A.; Wang A. Phyto-Mediated Synthesis of Ag Nanoparticles/Attapulgite Nanocomposites Using Olive Leaf Extract: Characterization, Antibacterial Activities and Cytotoxicity. Inorg. Chem. Commun. 2023, 151, 110543 10.1016/j.inoche.2023.110543.

[ref56] Casas L.; Mantell C.; Rodríguez M.; Torres A.; Macías F.; Martínez de la Ossa E. Supercritical Fluid Extraction of Bioactive Compounds from Sunflower Leaves with Carbon Dioxide and Water on a Pilot Plant Scale. J. Supercrit. Fluids 2008, 45 (1), 37–42. 10.1016/j.supflu.2007.12.002.

[ref57] Achat S.; Tomao V.; Madani K.; Chibane M.; Elmaataoui M.; Dangles O.; Chemat F. Direct Enrichment of Olive Oil in Oleuropein by Ultrasound-Assisted Maceration at Laboratory and Pilot Plant Scale. Ultrason. Sonochem. 2012, 19 (4), 777–786. 10.1016/j.ultsonch.2011.12.006. 22281378

[ref58] Escobar-Avello D.; Mardones C.; Saéz V.; Riquelme S.; von Baer D.; Lamuela-Raventós R. M.; Vallverdú-Queralt A. Pilot-Plant Scale Extraction of Phenolic Compounds from Grape Canes: Comprehensive Characterization by LC-ESI-LTQ-Orbitrap-MS. Food Res. Int. 2021, 143, 110265 10.1016/j.foodres.2021.110265. 33992366

[ref59] Fernández-Ponce M. T.; Parjikolaei B. R.; Lari H. N.; Casas L.; Mantell C.; Martínez de la Ossa E. J. Pilot-Plant Scale Extraction of Phenolic Compounds from Mango Leaves Using Different Green Techniques: Kinetic and Scale up Study. Chem. Eng. J. 2016, 299, 420–430. 10.1016/j.cej.2016.04.046.

[ref60] Rajapaksha S.; Shimizu N. Pilot-Scale Extraction of Polyphenols from Spent Black Tea by Semi-Continuous Subcritical Solvent Extraction. Food Chem. X 2022, 13, 100200 10.1016/j.fochx.2021.100200. 35498997 PMC9039883

[ref61] Erragued R.; Braga M. E. M.; Bouaziz M.; Gando-Ferreira L. M. Integration of Solvent Extraction and Membrane Processes to Produce an Oleuropein Extract from Olive Leaves. Sep. Purif. Technol. 2022, 299, 121751 10.1016/j.seppur.2022.121751.

