# Supplementary material for: Guide for Optimization of Olive Leaf Extraction and Silver Nanoparticles Biosynthesis as an Initial Step for Pilot Plant Design
Source: ACS Omega. 2024 Jun 17;9(26):29053–68. doi: 10.1021/acsomega.4c04483 (PMC11223521; doi:10.1021/acsomega.4c04483)
Supplement: Supplementary file 1 — ao4c04483_si_001.pdf [file ao4c04483_si_001.pdf]

Supplementary material

Guide for optimization of olive leaf extraction and AgNPs biosynthesis as an initial step for pilot plant design

Anna Wirwis[a], Zygmunt Sadowski[a*]

[a]*Department of Process Engineering and Technology of Polymer and Carbon Materials*, Faculty of Chemistry, Wrocław University of Science and Technology, Wybrzeze Wyspianskiego 27, 50-370 Wrocław, Poland.

E-mails: anna.wirwis@gmail.com; zygmunt.sadowski@pwr.edu.pl;

***Correspondence to**: Anna Wirwis

E-mail address: anna.wirwis@gmail.com

Telephone: +48 71 320 29 75

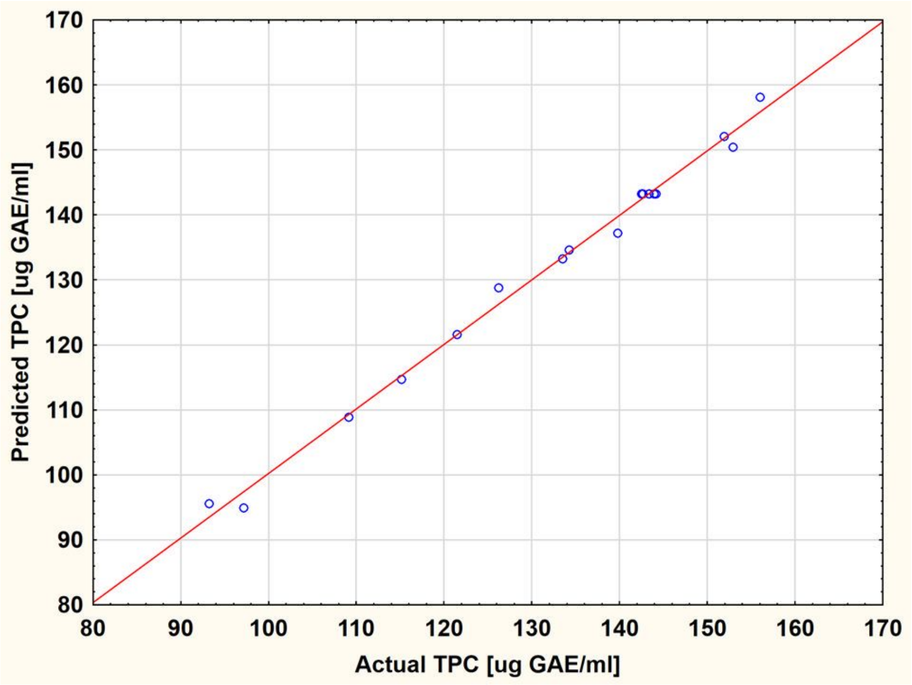

Figure S1. Linear relationship between the actual and predicted value of TPC as a result of extraction of olive tree L.

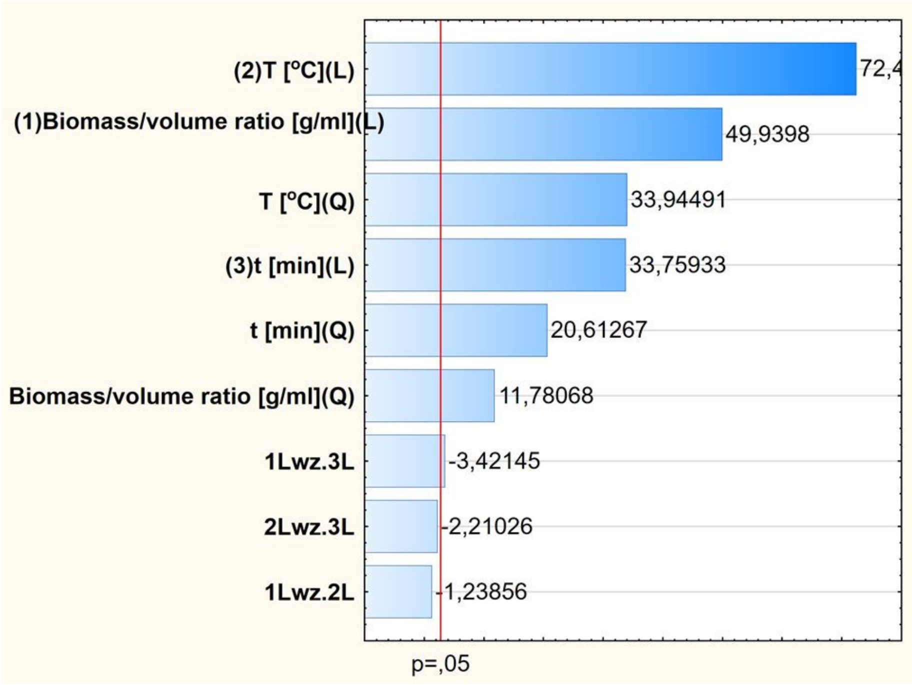

Figure S2. Pareto graph of the standardized effect of independent variables and interaction between them for the aqueous extraction of olive tree L.

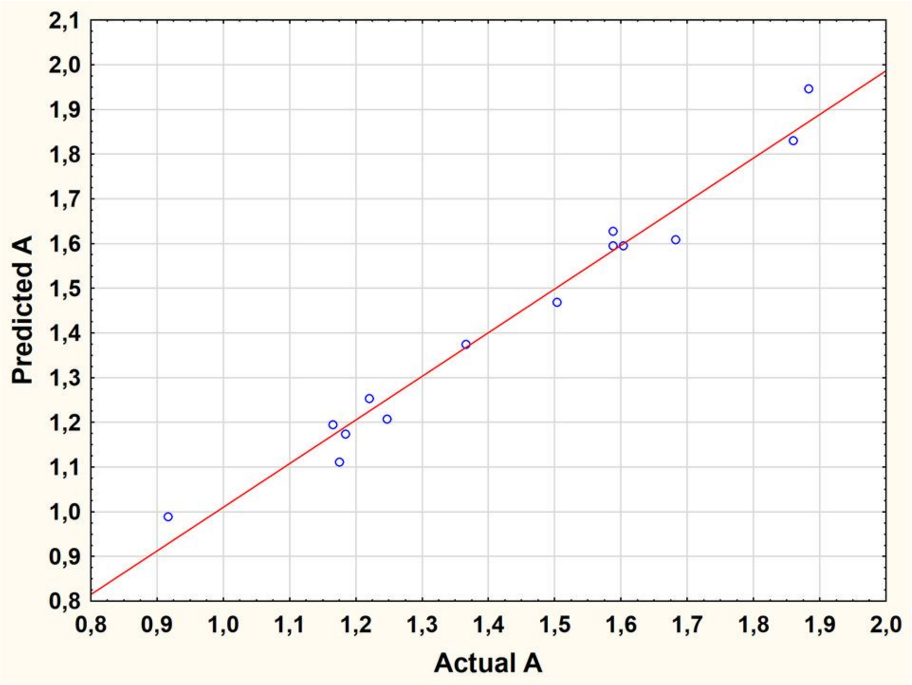

Figure S3. Linear relationship between the actual and predicted value of A as a response to the biosynthesis of AgNPs by olive tree L extract.

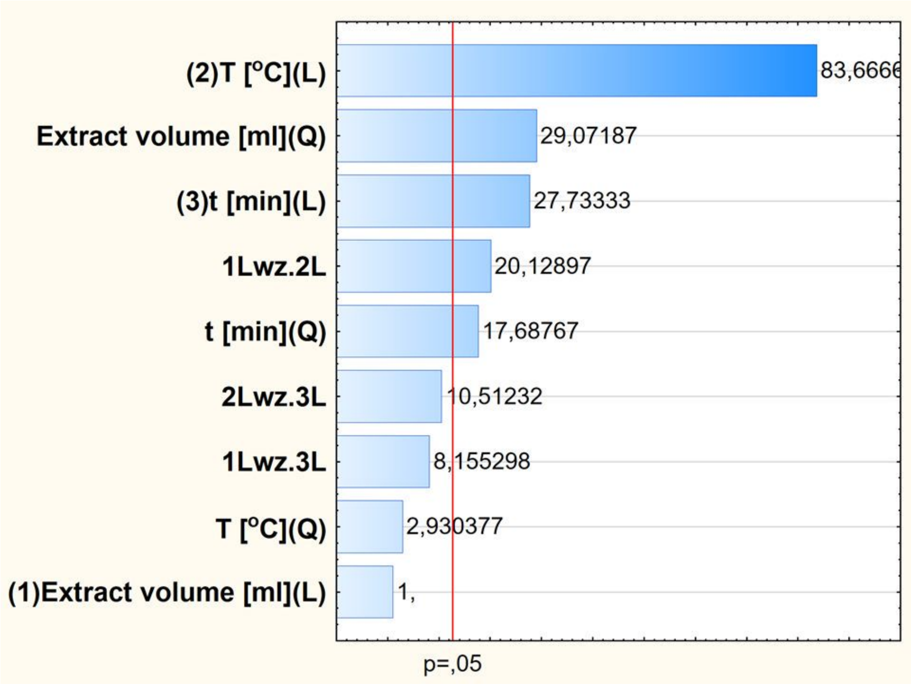

Figure S4. Pareto graph of the standardized effect of independent variables and interaction between them for the yield of biosynthesis of AgNPs.

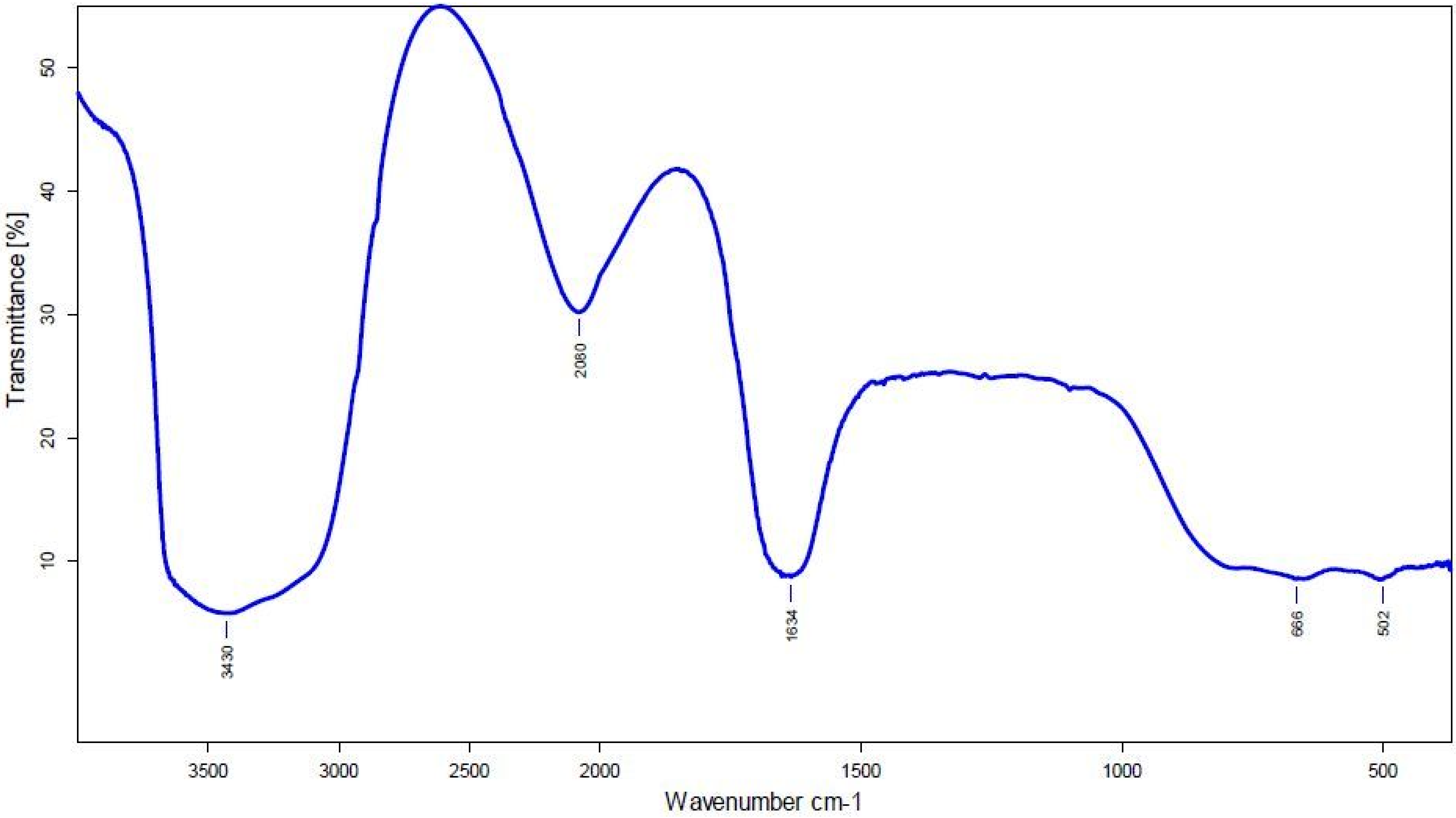

Figure S5. FTIR spectra for aqueous *Olea europaea* L. extract.

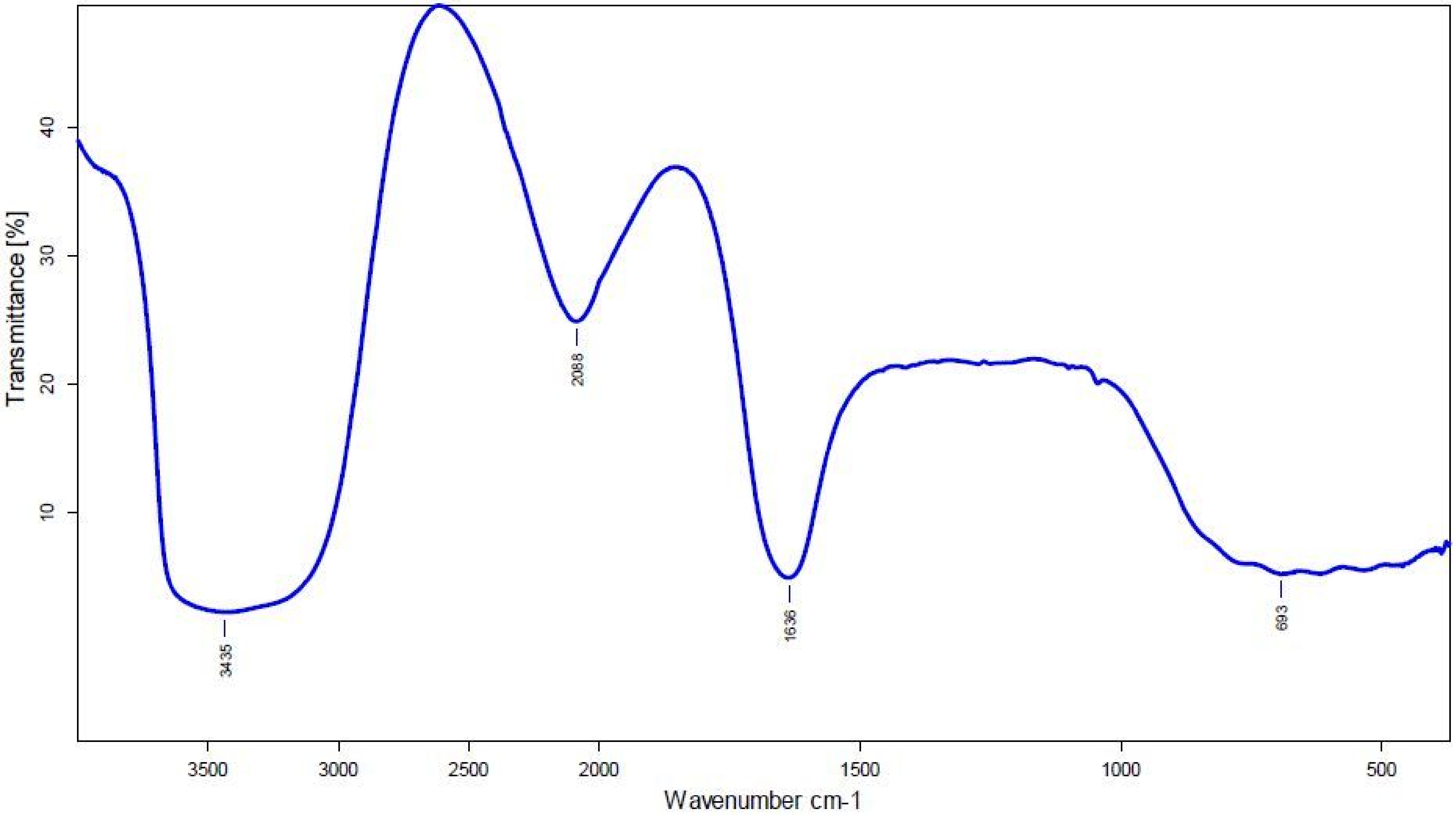

Figure S6. FTIR spectra for sample after biosynthesis of AgNPs using aqueous *Olea europaea* L. extract.
